# Bruceine H Mediates EGFR-TKI Drug Persistence in NSCLC by Notch3-Dependent β-Catenin Activating FOXO3a Signaling

**DOI:** 10.3389/fonc.2022.855603

**Published:** 2022-04-08

**Authors:** Jiahui Wu, Xiao He, Ziwei Xiong, Lingyu Shi, Daofeng Chen, Yulin Feng, Quan Wen

**Affiliations:** ^1^ Pharmacy, Jiangxi University of Chinese Medicine, Jiangxi, China; ^2^ National Pharmaceutical Engineering Center for Solid Preparation in Chinese Herbal Medicine, Jiangxi, China; ^3^ Pharmacy, Fudan University of Pharmacy, Shanghai, China

**Keywords:** bruceine H, Notch3 inhibitor, EGFR-TKI, acquired resistance, non-small cell lung cancer

## Abstract

Tyrosine kinase inhibitors (TKIs) targeting epidermal growth factor receptor (EGFR) protein serve as a critical pillar in the treatment of non-small cell lung cancer (NSCLC), but resistance is universal. Identifying the potential key factors of drug resistance to EGFR-TKIs is essential to treat patients with EGFR mutant lung cancer. Our research here shows that bruceine H suppressed the proliferation, migration, and invasion of lung cancer cells; inhibited the growth of human NSCLC cell xenografts; and enhanced the therapeutic effects of gefitinib in the PC-9/GR xenograft models, possibly by inhibiting Notch3. In order to analyze the potential targets of the combination of Notch3 and EGFR-TKIs on resistance to EGFR, we analyzed the differences of gene expression between NSCLC tissues and EGFR-driven gefitinib-resistant tumoral groups and then identify through the WGCNA key genes that may provide therapeutic targets for TKI-resistant lung cancer xenograft models. We confirmed that EGFR-TKI in combination with Notch3 inhibitor can inhibit the expression of β-catenin and enhance the level of FOXO3a, leading to improved recurrence-free survival and overall survival of the xenotransplantation model. These results support that the combination of gefitinib and bruceine H may provide a promising alternative strategy for treating acquired EGFR-TKI resistance in patients with NSCLC.

## Introduction

Lung cancer leads to more cancer deaths than breast, prostate, colorectal, and brain cancers grouped together (1). About a quarter of cancer deaths are caused by lung cancer. Lung cancer is classified into small cell lung cancer (SCLC) and non-small cell lung cancer, among which NSCLC cases account for about 83% of lung cancer, including squamous cell carcinoma, adenocarcinoma, and large cell lung cancer (2). Since there are no symptoms or no obvious symptoms in the early stage of NSCLC, most patients are diagnosed at stage IIIB or even stage IV at their first diagnosis, at which time the surgical treatment is not effective and the risk of recurrence is high, so medical treatment is mainly applied (3). The low survival rate of NSCLC patients reflects that most of them (57%) are diagnosed with drug resistance and metastatic diseases, and their 5-year relative survival rate is 23% (1, 4). In recent years, with the renewal of treatment methods, the improvement of chemotherapeutic agents, and the diversification of therapeutic strategies, the clinical therapeutic effect of lung cancer is obvious to all, but the drug resistance, metastasis, and low long-term survival rates are still critical challenges.

*Brucea javanica* (L.) Merr. is known to contain a large number of quassinoids, some of which possess a wide spectrum of biological activities, including anticancer (5), cancer chemopreventive (6), and cytotoxic activities (7). Previous studies showed that bruceine D (BD), a Notch inhibitor that can be derived from *B. javanica* seeds, exerts remarkable anticancer efficacy against human malignancies by Wnt/β-catenin (8) and MAPK pathways (9). Moreover, BD or brusatol can significantly increase the sensitivity of sorafenib or gefitinib to TKI-resistant tumors (8, 10). Although many studies have reported the antitumor activity of BD, it also showed certain cytotoxic activity to a normal human cell line (**Figure S1**). Bruceine H (BH) had only one more hydroxyl substitution at C-13 in its structure compared with bruceine D, improving its aqueous solubility and reducing its toxicity to normal cells.

Gefitinib is the first EGFR tyrosine kinase selective inhibitor (EGFR-TKI), which shows a fine curative effect on recurrent or advanced NSCLC (11). Lung cancer patients, who have undergone treatment for a few months, have been apparently resistant to gefitinib. Somatic activating mutations of EGFR, including the L858R mutation, G719X mutation, and deletion of exon 19 (12), are related to the sensitivity to EGFR-TKIs. In addition to EGFR mutations accounting for acquired resistance mechanisms, T790M site mutation (13); MET amplification and HER2 hyperactivation (14); fibroblast growth factor receptor (FGFR) (15), KRAS (16), PIK3CA, and B-Raf proto-oncogene (BRAF) V600E mutations (17); overexpression of EGF (18); and activation of MAPK (19) may also contribute to the EGFR-TKI acquired resistance. Increasing studies have reported that Notch inhibitor mediates EGFR-TKI drug persistence in the EGFR mutant NSCLC (20, 21).

The Notch signaling pathway is associated with the regulation of cellular proliferation, differentiation, and apoptosis during embryonic development and whole adulthood (22). Although the normal Notch signaling plays an important role in lung development and formation, especially in the transformation of lung plasticity and repair, its abnormal activity has been demonstrated to be related to the occurrence and progression of lung cancer, including NSCLC (23, 24). Twenty years ago, the discovery that Notch could be first implicated as a catalyst for NSCLC pathogenesis was made when a translocation of a somatic chromosome t (15, 19), resulting in overexpression of the Notch3 gene, was found in poorly differentiated and invasive lung adenocarcinoma (25). Recent findings indicate that Notch3 overexpression not only is responsible for the initiation of non-small cell lung cancer (26, 27), but also can reduce the expression of E-cadherin, upregulate fibronectin, and promote EMT (28) and tumor invasion (29). Therefore, inhibiting Notch3 could potentially make EGFR-TKI-resistant NSCLC more susceptible. Furthermore, the Notch3-dependent β-catenin signal has been shown to contribute to drug resistance associated with the second mutation of EGFR (20), while β-catenin binding to FOXO3a regulates the inhibitory effect of FOXO3a on EMT (30), which we speculate that Notch3-dependent β-catenin and FOXO3a signaling pathways possibly have a hand in resistance to EGFR-TKIs.

In this study, bruceine D and H, characterized by the core structure of tetracyclic triterpene, were extracted from *B. javanica*. We found that both of them are bound to Notch3 protein with a high affinity in computational modeling. Our *in-vitro* and *in-vivo* model system of gefitinib-induced drug-persistent cells (DPCs) has demonstrated Notch3 as a critical mediator of this effect, but the precise mechanisms by which Notch3 maintains specific targeting of this pathway are not understood. For this reason, we sought to identify differentially expressed genes (DEGs) and study the interactions among them in gefitinib-resistant lung cancer to identify drug-resistant core genes and drug targets. The experimental results show that Notch3 inhibition might be a potent strategy to treat patients with drug resistance and tumor recurrence of NSCLC.

## Materials and Methods

### Compounds and Reagents

*Brucea javanica* seeds were purchased from Guangxi Zhuang Autonomous Region, People’s Republic of China, and authenticated by Prof. Guo-Yue Zhong (Jiangxi University of Chinese Medicine). BD and BH were extracted from the seeds of *B. javanica* and identified on the basis of nuclear magnetic resonance (NMR) data (the separation method is described in detail in the **Supplementary Material**); their purity was ≥95% based on high-performance liquid chromatography (HPLC) analysis. Gefitinib was provided by Meilun (ZD1839, Dalian, China). The primary antibodies NOTCH3 (mouse), β-catenin (mouse), EGFR, phospho-EGFR (Tyr1068), FOXO3a (mouse), CBP (Lys1535)/p300 (Lys1499), and β-actin were from Cell Signaling Technology (CST, Danvers, MA, USA). Bax, Bcl-2, caspase-3, and cleaved caspase-3 were provided by Santa Cruz Biotechnology (SCBT, Dallas, TX, USA). The secondary antibodies were HRP-conjugated anti-rabbit IgG and anti-mouse IgG (Abcam, Cambridge, UK).

### Cell Lines and Cell Culture

The human NSCLC cell lines (A549, PC-9, and PC-9/GR) and other cell lines (16HBE and LO2) were purchased from the American Type Culture Collection (ATCC, Manassas, VA, USA). A549 and LO2 cells were cultured in RPMI-1640 (Solarbio, Beijing, China) containing 10% fetal bovine serum (FBS, Gibco, Carlsbad, CA, USA), and PC-9 and 16HBE cells were cultured in DMEM (Solarbio) with 10% FBS. The PC-9/GR cells, a gefitinib-resistant strain of human lung adenocarcinoma cell, were grown in DMEM with 10% FBS and gefitinib (800 ng/ml). All cell lines were cultivated at 37°C under 5% CO_2_.

### Cell Viability Assay

The Cell Counting Kit-8 assays (Beyotime, Shanghai, China) were performed to assess cell viability. A549 (5 × 10^3^/100 μl/well), PC-9 (6 × 10^3^/100 μl/well), or PC-9/GR cells (6 × 10^3^/100 μl/well) were cultured overnight in 96-well plates (NEST, Wuxi, China) and were then treated with several concentrations of BD, BH, and/or gefitinib at the indicated concentration for 24 or 48 h. Then, the cells were incubated for an additional 2 h with 100 μl of RPMI-1640/DMEM and 10 μl of CCK-8 solution at 37°C. Microplate readers (Infinite F500, Tecan, Mannedorf, Switzerland) were used to measure the absorbance (*A*) at 450 nm. All samples were assessed in triplicate.

### Wound Healing Assay for Migration

A549 cells (3 × 10^5^/ml/well) were seeded into a six-well plate. After overnight culture, when the fusion rate reached 95%–100%, the sterile pipette tip was perpendicular to the plate plane, and a straight wound was drawn on the cell monolayer. Following treatment with BD or BH, the migration of the cells was examined under a microscope (×4, Nikon, Tokyo, Japan), and pictures were obtained at 0 and 48 h. The computation of the cell migration formula is as follows: relative migration area (%) = (0 h wound area – 48 h wound area)/0 h wound area × 100%.

### Transwell Migration and Matrigel Invasion Assays

The Transwell insert in a 24-well plate (8 μm, Corning, NY, USA) was taken out, and a 600-μl medium containing 10% FBS was added to the bottom chamber. The A549 cells were resuspended with 10% FBS culture medium, and the diluted cell suspension (3 × 10^5^/300 μl) was cultured in the top chamber and then incubated with or without Matrigel (BD BioCoat). After 12 h, the medium in the top chamber was changed to RPMI-1640 without FBS, and BD or BH with different concentrations was added (31). After an additional 12 h, the cells that migrated or invaded the bottom side of the polycarbonate film were fixed with methanol for 30 min and then stained for 15 min with 0.1% crystal violet (Solarbio), after which their number was assessed under a microscope (×4, Nikon).

### Apoptosis Analysis by Flow Cytometry

Cells (4 × 10^5^) were seeded in six-well plates, incubated overnight, and treated for 48 h with BH or gefitinib alone or combined before staining before examining with an Annexin V-FITC Apoptosis Detection Kit (Yeasen, Shanghai, China). After being digested with trypsin without EDTA, the cells were washed gently with culture medium and twice with cold PBS. The pellet was resuspended in 400 μl of 1× binding buffer, the resuspended cells were transferred to flow tubes, then Annexin V-FITC (5 μl) and propidium iodide (10 μl) were added, and the mixture was incubated for 15 min at room temperature in the dark. The percentage of apoptotic cells was determined by flow cytometry (Backman, CA, USA) and FlowJo V10 software.

### Docking Analysis

The ligand molecule was downloaded from the PubChem database (https://pubchem.ncbi.nlm.nih.gov/) and it was further converted to a PDB file using Open Babel GUI. The crystal structures of EGFR and Notch3 corresponding to PDB ID (3G5Z, 4ZLP) were obtained from the PDB database (https://www.rcsb.org/). The crystal water and other small molecules were removed from the protein structure by PyMOL (https://pymol.org), then hydrogens and charges were added using the AutoDock tool (http://autodock.scripps.edu/). Discovery Studio (http://dstudio19.csc.fi:9944) was used to predict the hydrophobic interaction between BD, BH, and gefitinib with the active site of the residue, along with the force of binding.

### Identification of Potential Resistance Targets in NSCLC Based on Weighted Gene Co-Expression Network Analysis and Differential Gene Correlation Analysis

The Gene Expression Omnibus (GEO) database (https://www.ncbi.nlm.nih.gov/geoprofiles/) was searched to download two microarray datasets (GSE115864 and GSE33532), and then their gene expression level and differential expression were analyzed. The detailed clinical information and complete mRNA expression profile data of 552 lung adenocarcinoma (LUAD) samples and 504 lung squamous cell carcinoma (LUSC) samples were obtained from The Cancer Genome Atlas database (TCGA, https://tcga-data.nci.nih.gov/tcga/) (accessed on May 12, 2021). We used the limma (linear models for microarray data) tool (32) to screen DEGs between PC-9 cells resistant to gefitinib and PC-9/GR treated with Notch3 inhibition and gefitinib, overlapped with the most significant module identified by weighted gene co-expression network analysis (WGCNA) (33) to obtain the hub genes, and then we subsequently carried out Gene Ontology (GO) functional annotation analysis and Kyoto Encyclopedia of Genes and Genomes (KEGG) pathway enrichment analysis. Then, the key genes were predicted by Cytoscape plugin cytoHubba, and six key genes were selected according to the expression patterns in GEO and TCGA databases. Finally, the diagnostic value of the top six key genes was further verified by survival analysis and expression level in mutant lung cancer.

### Western Blotting

The total proteins of cells and tissues were extracted with RIPA cleavage buffer containing a protease inhibitor cocktail, phenylmethylsulfonyl fluoride (PMSF, Solarbio). The cell lysates (20 μg) were run on SDS-polyacrylamide gel (6%–10%) and transferred to the polyvinylidene fluoride membrane (PVDF, Millipore, MA, USA) by electrophoresis. The membrane was blocked with 5% skim milk in TBST for 2 h, incubated overnight with the corresponding primary antibody at 4°C, and then incubated with the corresponding secondary antibody at room temperature for 2 h. The reaction products were detected by chemiluminescence by ECL Western Blotting Detection System. Finally, quantitative analysis was carried out with ImageJ software.

### H&E and TUNEL Staining

The heart, liver, spleen, lung, kidney, and tumor tissues were embedded in paraffin, and the sections (4 µm) were stained with hematoxylin–eosin (H&E, Solarbio) and then examined and analyzed under a Leica DM6B microscope (Leica Microsystems, Wetzlar, Germany).

A TUNEL Assay Kit (Yeasen) was used to detect apoptosis. Briefly, the tumor section was dewaxed and incubated with proteinase K at 37°C for 20 min, followed by incubation with labeling buffer containing terminal deoxynucleotidyl transferase (TdT) and digoxigenin-labeled deoxyuridine triphosphate (dUTP). Then, the tissues were stained with 4ˊ,6-diamidino-2-phenylindole (DAPI) to stain the cell nucleus. TUNEL-positive cells were quantified by counting positively stained cells.

### Immunocytochemistry and Immunohistochemistry

The PC-9/GR cells were added to glass slides in six-well plates and treated with BH and gefitinib in different concentrations, and DMSO served as an untreated control. The primary FOXO3a antibody (CST) was diluted at 1:100 and incubated at 4°C overnight. Afterward, the goat anti-rabbit IgG secondary antibody (dilution ratio: 1:400; Abcam) was added to the fixed PC-9/GR cells in a dark condition for 1 h and the nuclei were stained with DAPI (CST) for 5 min. Finally, fluorescence images were captured using the LAS AF Lite software and Leica DM6B microscope (Leica Microsystems).

The tumor section was dewaxed and repaired with antigen, 3% H_2_O_2_ was added, and then the section was sealed with 10% goat serum at room temperature for 30 min. The first antibody against Ki-67 and Notch3 (CST) was incubated overnight at 4°C, and the second antibody was incubated at room temperature for 30 min. After DAB staining, the sections were counterstained with hematoxylin for 2 min and then observed and analyzed under a Leica microscope.

### Tumor Xenografts

The animal procedures were approved by the Ethics Committee of the Experimental Animal Center of Jiangxi University of Chinese Medicine (Nanchang, China, SCXK 2016-0006) and conducted following the Guide for the Care and Use of Laboratory Animals. Four-week-old male BALB/c nude mice (weighing 16–17 g, SPF grade) were obtained from Shanghai Shrek Experimental Animal Co., Ltd. (Shanghai, China). The xenotransplantation model was established by subcutaneous injection of A549 or PC-9/GR cells in nude mice, which were collected with PBS and mixed with an equal volume of Matrigel at a final concentration of 1 × 10^7^/ml. Approximately 100 mm^3^ of tumor volume was reached, and mice were randomly divided into four groups: vehicle control, gefitinib alone, BH alone, or a combination of gefitinib and BH. Gefitinib (25, 50 mg/kg) and BH (2.5, 5 mg/kg) were dissolved in 0.5% CMC-Na containing 5% DMSO and administered every day by oral gavage. Measurements of tumor size and body weight were conducted every 2 days with a vernier caliper and an electronic balance. Nodule growth was quantified by using the following formula: tumor volume (mm^3^) = (short-diameter)^2^ × large-diameter × π/6.

### Toxicity and Pharmacokinetic Study

At the end of the experiment, blood samples were taken and analyzed by blood routine. The remaining blood samples were precipitated, followed by a 15-min centrifugation (3,000 rpm) at 4°C, and the serum was taken for biochemical analysis. The lungs and spleen were weighed and recorded. H&E staining of the heart, liver, spleen, lungs, and kidneys was performed to evaluate the cytotoxic effects of BH.

We administered BH by gavage and intravenous administration to SD rats (male, 200–220 g, *n* = 6) after they had fasted for 12 h. For oral gavage, the BH was dissolved in DMSO/0.5% CMC-Na (5/95, v/v/), and for intravenous administration, it was dissolved in DMSO/0.9% NaCl (5/95, v/v/), and then the blood samples were collected at intervals to determine the pharmacokinetic (PK) profiles of SD rats.

### Statistical Analysis

All the experimental data were analyzed by GraphPad Prism 8.0 software (USA). The results were expressed as the average ± SEM of at least three independent experiments. Double-tailed *t*-test and ANOVA were used; when *P <0.05, **P <0.01, or ***P <0.001, it was considered to be statistically significant.

## Results

### The Structure of the Notch Inhibitors BH and BD

**Figure 1:**
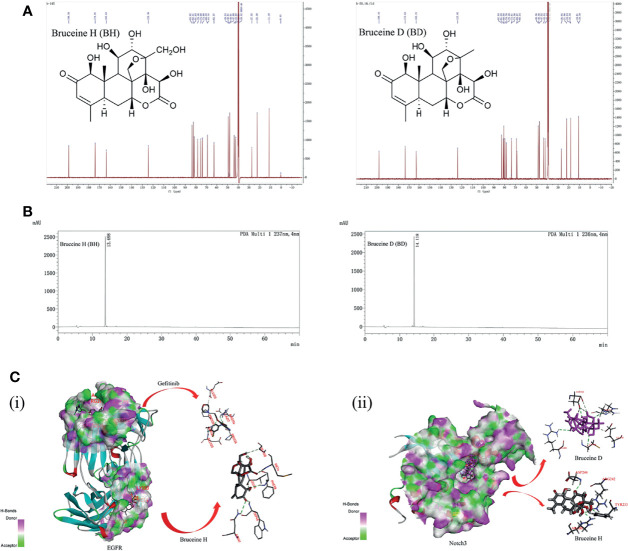
The interaction of bruceine H (BH) with Notch3 and epidermal growth factor receptor (EGFR) proteins. **(A)** Chemical structure of BH and bruceine D (BD) by ^13^C NMR spectrum. **(B)** The purity of BH and BD by HPLC chromatogram. **(C)** Computational modeling of BH binding to Notch3 and EGFR. (i) 3D interaction of BH and gefitinib with the EGFR protein. (ii) 3D interaction of BH and BD with the Notch3 protein.

The structures of BH and BD are presented in **Figure 1A**, which were identified by ^1^H and ^13^C nuclear magnetic resonance (NMR) (**Figures S6–S9**). HPLC analysis was performed in our study to confirm that the purity of BH and BD was ≥95% (**Figure 1B**). Bruceine H and gefitinib showed very good binding affinity (−31.9, −20.0 kcal/mol) with the amino acid residues PHE98, TRP48 and GLU123, ARG216 of the target protein EGFR (3G5Z), respectively. Furthermore, the Notch inhibitors BH and BD were also docked to find the interaction and binding affinity (LibDockScore:169.0, 165.2 >120) with the target protein Notch3 (4ZLP). BH and BD bind to the same sites on the Notch3 HD domain in modeling, despite the different chemical formula (**Figure 1C**). This shows that both of these compounds have similar affinity to Notch3 protein, and in the subsequent study of the cell model, BH and BD have also been proven to have similar antitumor activity.

### BH Significantly Suppressed Migration and Invasion and Induced Apoptosis in NSCLC Cells

**Figure 2:**
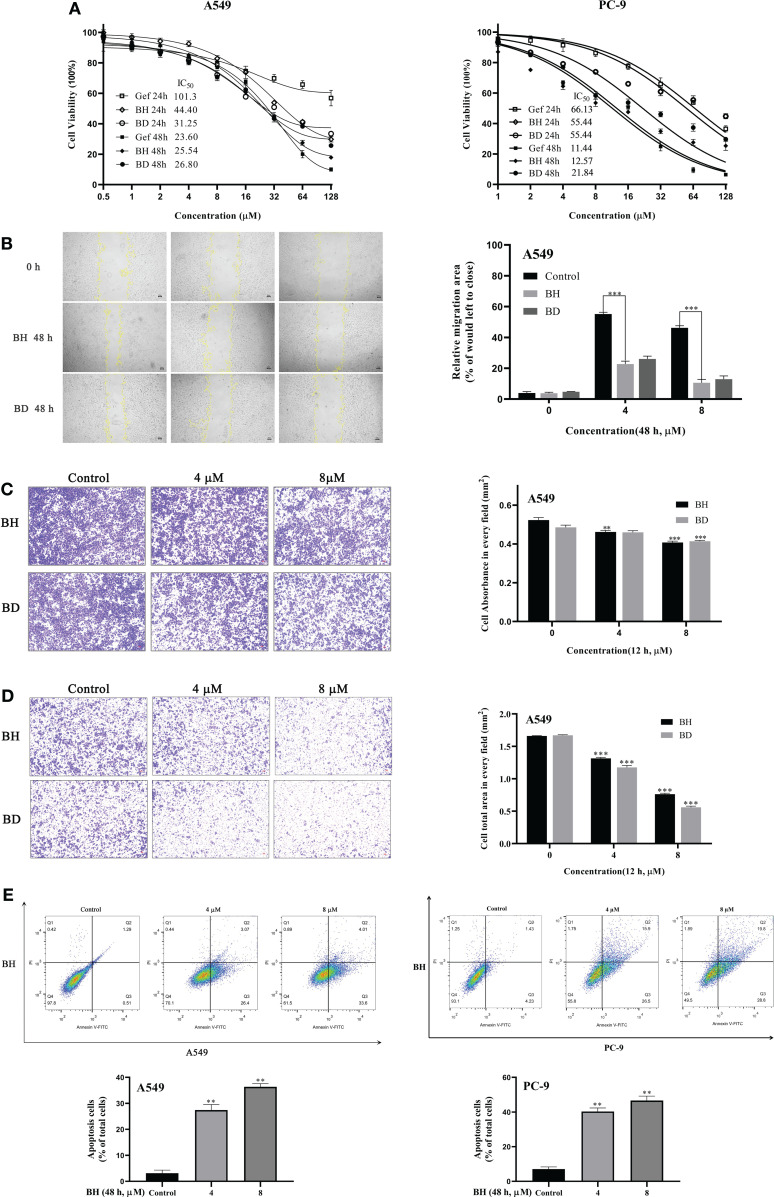
Antitumor effects of BH and BD against non-small cell lung cancer (NSCLC) cell lines *in vitro*. **(A)** The proliferation of cells as well as the IC_50_ was determined in A549 and PC-9 cells after 24 and 48 h. **(B)** Cell migration of A549 cells was detected by wound healing assay. **(C, D)** Transwell migration and Matrigel invasion assays of A549 cells were measured. **(E)** The apoptosis of A549 and PC-9 cells was measured using flow cytometry 48 h after treatment with BH. All experiments were independently conducted at least three times and showed representative data. “**” represents BH group vs. the Control group (**p < 0.01, ***p < 0.001).

The NSCLC cell lines A549 (EGFR wild-type KRAS mutation) and PC-9 (EGFR exon 19 deletion) were treated with BH and BD at different concentrations for 24 and 48 h, respectively. The results showed that gefitinib, BH, and BD could inhibit the proliferation of A549 and PC-9 cells in a dose-dependent manner. The IC_50_ (48 h) values of gefitinib, BH, and BD against A549 cells were 23.60, 25.54, and 26.80 μM, and the IC_50_ values against PC-9 cells were 11.44, 12.57, and 21.84 μM, respectively (**Figure 2A**). The inhibitory effect of BH on cell proliferation was similar to that of BD, especially on A549 cells.

Given the role that migration and invasion of cancer cells play in cancer metastasis and patient mortality, further investigation of BH and BD for their effect on migration and invasion was conducted. Wound healing assay showed that the migration of A549 cells was impaired by 4 μM BH (**Figure 2B**). Transwell cell invasion assay results demonstrated that BH and BD can exert significant anti-invasive and antimigratory actions toward A549 in a dose-dependent manner (**Figures 2C, D** **)**. In an attempt to determine whether the proliferative inhibition by BH was related to apoptosis, cells from A549 and PC-9 were treated with BH for 48 h to determine the percentage of apoptotic cells. **Figure 2E** shows that the proportion of apoptotic cells increased after treatment with various concentrations of BH, as well as the early apoptosis rate in A549 cells, while the late apoptotic rate of PC-9 cells increased, relative to the control conditions. It is noteworthy that BD shows higher sensitivity in 16HBE and LO2 cell lines than BH (**Figure S1**). Taken together, BH exhibited not only potent antitumor activities but also no apparent toxicity to normal human cell lines and was selected for further studies. Then, we further determined whether BH would synergize with gefitinib to decrease the PC-9/GR cell viability.

### BH Combined With Gefitinib Inhibited Gefitinib-Resistant Cell Lines PC-9/GR and Notch3 Signaling

**Figure 3:**
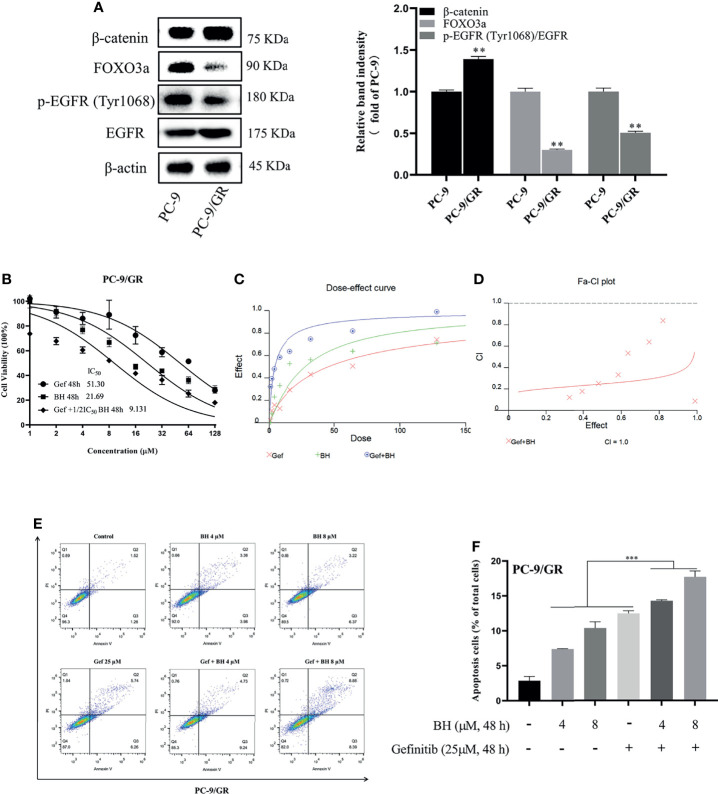
BH significantly enhanced gefitinib sensitivity *in vitro*. **(A)** The different expressions of β-catenin, FOXO3a, p-EGFR (Tyr1068), and EGFR in PC-9 and PC-9/GR cells were measured by Western blot. **(B)** PC-9/GR cells were incubated with BH, gefitinib, and combination at the indicated concentrations for 48 h. **(C, D)** The combined action curve of gefitinib and BH and the synergistic effect index of drug combination in PC-9/GR cells (CI = 1 when the two drugs were superimposed, CI < 1 when the combination of drugs appeared synergistic effect, CI > 1 when antagonistic effect appeared). **(E, F)** Representative flow cytometry of PC-9/GR cells treated with gefitinib alone or combined with BH for 24 h. “**” represents BH group vs. the Control group (**p < 0.01, ***p < 0.001).

Lung cancer with EGFR exon 19 deletion is known to have the greatest prevalence, which is present in 1.57% of AACR GENIE cases (34). As compared with PC-9 cells, gefitinib-resistant PC-9 cells (PC-9/GR) contain the same exon 19 deletions of the EGFR gene but show low sensitivity to gefitinib and lower expression of FOXO3a (**Figure 3A**). PC-9 cells harboring EGFR activation mutation were sensitive to gefitinib, IC_50_ at 11.44 μM, while PC-9/GR was resistant to gefitinib under long-term low concentration of gefitinib, IC_50_ at 51.30 μμ (**Figure 3B**). **Figures 3C, D** show that drug synergism in PC-9/GR was calculated using CalcuSyn 2.0 software when BH and gefitinib are combined according to their IC_50_ concentrations. It was discovered that drug synergy increased as drug concentration was increased, and the drug synergy was the strongest at an inhibition rate of 80%. As a result of gefitinib treatment alone, PC-9/GR is no longer sensitive to gefitinib, and BH and gefitinib combined improved the inhibitory effect compared with a single treatment. To explore the effects of BH and gefitinib on the apoptosis of PC-9/GR cells, the results of flow cytometry showed that low-dose BH or gefitinib alone had little effects, while a combination of BH and gefitinib obviously induced apoptosis in PC-9/GR cells compared with gefitinib monotherapy (**Figures 3E, F** **)**.

**Figure 4:**
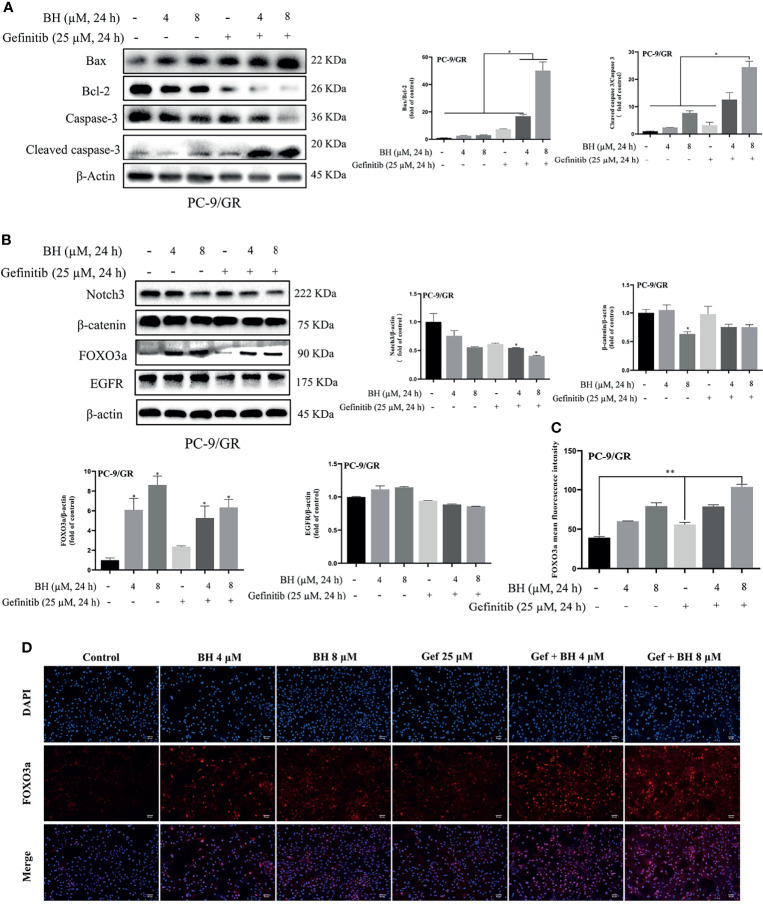
BH modulates the expression of multiple proteins. **(A)** Western bolt was used to detect the expression of cleaved caspase-3, caspase-3, Bcl-2, and Bax protein. **(B)** The protein levels of Notch3, β-catenin, FOXO3a, and EGFR in PC-9/GR cells treated with BH (4, 8 μM) and/or gefitinib (25 μM) for 24 h. **(C, D)** Immunofluorescent images of PC-9/GR cells represented the accumulation of FOXO3a. Scale bars, 50 μm. “*” represents Gef +BH group, BH group, Gef group vs. the Control group (*p < 0.05, **p < 0.01).

Subsequently, the extracts of PC-9/GR cells treated with BH and/or gefitinib for 48 h were prepared for immunoblotting. The Western blot analysis showed that BH combined with gefitinib treatment deregulated the anti-apoptotic protein (Bcl-2) and significantly increased the expression of Bax. Additionally, compared with the vehicle and gefitinib or BH alone, the expression of cleaved caspase-3 was remarkably increased and caspase-3 was inhibited (**Figure 4A**). Together, these results indicate that bruceine H in combination with gefitinib results in a significant induction of PC-9/GR apoptosis. Additionally, the level of FOXO3a was activated and β-catenin levels were suppressed by a combination of gefitinib and BH (**Figure 4B**). We found that, through inhibiting the activity of β-catenin and activating FOXO3a in drug-resistant cells, BH enhanced the sensitivity of PC-9/GR cells to gefitinib. To clarify the role of FOXO3a, we confirmed the increase of FOXO3a protein accumulation in PC-9/GR cells by immunofluorescence staining (**Figures 4C, D** **)**. These results indicated that BH significantly enhanced gefitinib sensitivity in PC-9/GR cells by β-catenin activating the FOXO3a signaling pathway.

### Identifying Hub Genes of EGFR-TKI Drug Persistence in EGFR Mutant by DEGs and WGCNA

**Figure 5:**
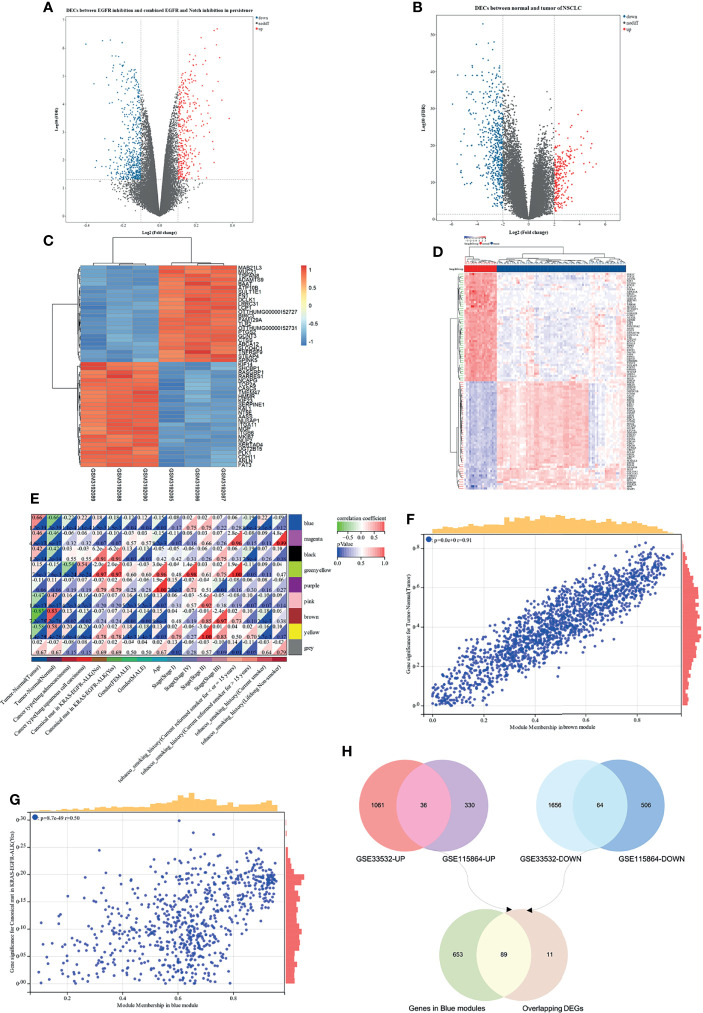
WGCNA and differentially expressed genes (DEGs) of EGFR-TKI drug persistence in EGFR mutant NSCLC. **(A)** Volcano maps of DEGs between two groups: EGFR inhibition and combined EGFR and Notch inhibition in EGFR mutant of NSCLC. **(B)** Volcano maps of DEGs between normal and tumor. Upregulated genes are shown in red and downregulated genes are shown in green dots. **(C, D)** Heatmap showing the expression level of the top 50 DEGs. **(E)** Each column represents a clinical feature and each row represents a module eigengene; red represents a positive correlation and green represents a negative correlation; the darker the hue, the higher the correlation. **(F, G)** Scatter plot of eigengene modules. **(H)** Venn diagram showed the intersection of overlapping DEGs of N-T DEGs and E-N DEGs and genes in blue modules.

Based on the analysis of the differentially expressed genes between EGFR inhibition and combined EGFR and Notch inhibition in EGFR mutant of NSCLC, a total of 936 DEGs were identified, consisting of 366 upregulated and 570 downregulated DEGs (**Figure 5A**). On the other hand, the difference of gene expression between 80 non-small cell lung cancer samples and 20 marginal samples yielded 2,817 DEGs, consisting of 1,097 high-expression genes and 1,720 low-expression genes (**Figure 5B**). **Figures 5C, D** represent the heatmap of the expression levels of these DEGs. The 259 NSCLC samples from the TCGA database (**Table S1**), with a clear record of a canonical mutation in KRAS, were used to establish a co-expression module for WGCNA to evaluate the correlation between potential genes and EGFR mutations in patients with non-small cell lung cancer. Cluster analysis demonstrated that the mutation in the sample has a certain relationship with smoking history, and the longer the smoking period, the more likely it is that the mutation of lung cancer would occur (**Figure S2A**). Besides, the power value of *β* = 4 (scale-free *R* ^2^ = 0.91) was set to ensure low mean connectivity and high-scale independence (**Figures S2B, C**). A total of nine modules (**Figures S2D, E**) were identified through the average linkage hierarchical clustering and segmented the clustering results under the set criteria (dissimilarity = 0.25). The correlation of the modules with clinical traits, including tumor-normal and EGFR-TKI mutant, is shown in **Figures 5E–G**. Finally, the 742 gene sequences in the blue module were intersected with mutant-specific DEGs to identify 89 genes that regulated drug persistence in patients with NSCLC with EGFR mutations (**Figure 5H**).

### GO Function and KEGG Pathway Annotation of Module Hub Genes

**Figure 6:**
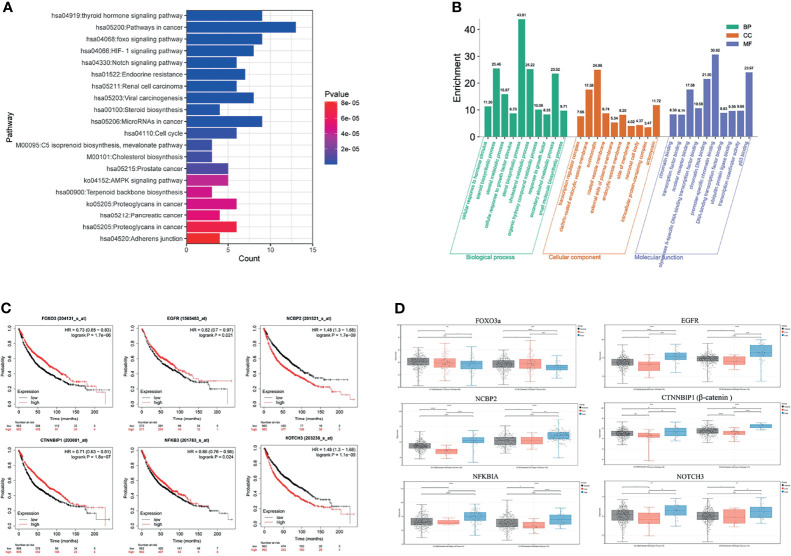
Function and pathway enrichment analysis and key gene cluster. **(A)** KEGG enrichment analysis. **(B)** GO enrichment analysis. **(C)** Kaplan–Meier survival curves of six prognostic biomarkers screened from the hub gene. **(D)** Contrast of the expression of the six hub genes in the EGFR mutant LUAD and LUSC dataset.

GO function and KEGG pathway enrichment analyses were performed to assess the function of 89 genes, which intersected the blue module genes and the two differential overlapping genes. The top 10 results of GO that include terms of biological process (BP), cellular component (CC), and molecular function (MF) are shown in **Figure 6A**. The most significant enrichment terms of BP, CC, and MF for EGFR-TKI drug persistence were “sterol biosynthetic process”, “clathrin-coated endocytic vesicle membrane”, and “nuclear receptor binding”, respectively. Twenty significantly enriched KEGG pathways were identified in these overlapping genes (**Figure 6B**). The most significant KEGG pathways, including the FOXO and Notch signaling pathways, might be related to the EGFR resistance response of NSCLC patients.

The survival analysis of the genes in the above network was conducted to determine the prognostic value of the six hub genes in NSCLC. In the relapse-free survival (RFS) analyses based on sequencing data of 1,925 NSCLC patients, high NOTCH3 and NCBP2 expression levels were associated with shorter NSCLC survival times (**Figure 6C**, *P* < 0.01). Furthermore, individuals expressing high levels of FOXO3a had a more favorable prognosis than those expressing low levels of the gene. We analyzed the expression levels of FOXO3a, EGFR, NCBP2, CTNNBIP1, NFKBIA, and NOTCH3 at different resistance statuses in LUAD and LUSC in the TCGA database (**Figure 6D**). The result indicated that the Notch3 and β-catenin genes were overexpressed in EGFR resistance processes in NSCLC.

### BH Inhibited the Growth of Human Lung Cancer Cell Xenografts

**Figure 7:**
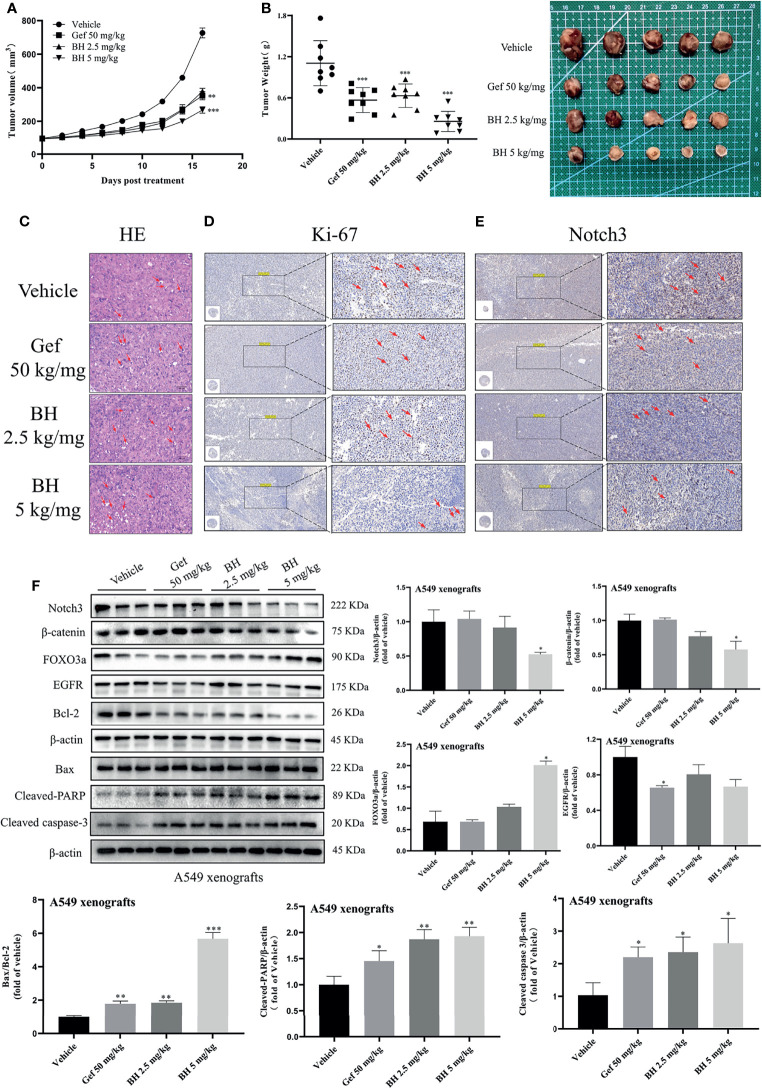
BH inhibits the growth of lung cancer xenografts in BALB/c athymic nude mice. **(A, B)** Tumor volume and tumor weight. **(C)** H&E staining of tumor tissues, and the scale bar was 100 μm. **(D, E)** IHC was used to detect the protein levels of Ki-67 and Notch3 of A549 xenografts, and the scale bar was 200 μm. **(F)** Protein levels of Notch3, β-catenin, FOXO3a, EGFR, Bcl-2, Bax, cleaved caspase-3, and cleaved-PARP in tumor samples were determined by Western blot. “*” represents Gef +BH group, BH group, Gef group vs. the group (*p < 0.05, **p < 0.01, ***p< 0.001).

Subcutaneous mouse xenografts of the NSCLC cell line A549 were further used to evaluate BH’s antitumor effect. BH significantly inhibited the growth of human lung cancer (A549) xenografts after 16 days of treatment with 2.5 or 5 mg/kg dose given *via* intragastric administration, and the inhibitory effect of 2.5 mg/kg brucine H was similar to that of the effective drug gefitinib (**Figures 7A, B** **)**. For A549 xenograft tumors, tumor volumes and tumor weights decreased dose-dependently in the 2.5- and 5-mg/kg treatment groups (**Figure S3A**) without significant changes in body weight. Meanwhile, there were no significant changes in organ indexes of the heart, kidney, liver, lung, and spleen tissues (**Figure S3B**), indicating that BH treatment did not cause harmful side effects in tumor-bearing mice. Moreover, tumor cell apoptosis was evaluated by HE staining, and the mitotic index (Ki-67) and Notch3 signal were detected through immunohistochemistry (IHC) and Western blotting. In contrast to the vehicle group, BH caused significant cancer cell apoptosis, and the number of Ki-67-positive cells in cancer tissues was decreased (**Figures 7C, D, F** **)**. Notch3 expression in tumor xenografts decreased in a dose-dependent manner in the BH treatment group compared with that in the model group, according to the IHC analysis (**Figure 7E**). Additionally, in the BH treatment group, the expression of FOXO3a increased in the tumor tissues, whereas Notch3, β-catenin, and Bcl-2 protein levels decreased (**Figure 7F**). The results showed that BH significantly suppressed excessive Notch3 signaling and displayed potent antitumor activity against human lung cancer cell xenograft models.

### BH Enhanced the Antitumor Effects of Gefitinib in TKI-Resistant Lung Cancer Xenografts

**Figure 8:**
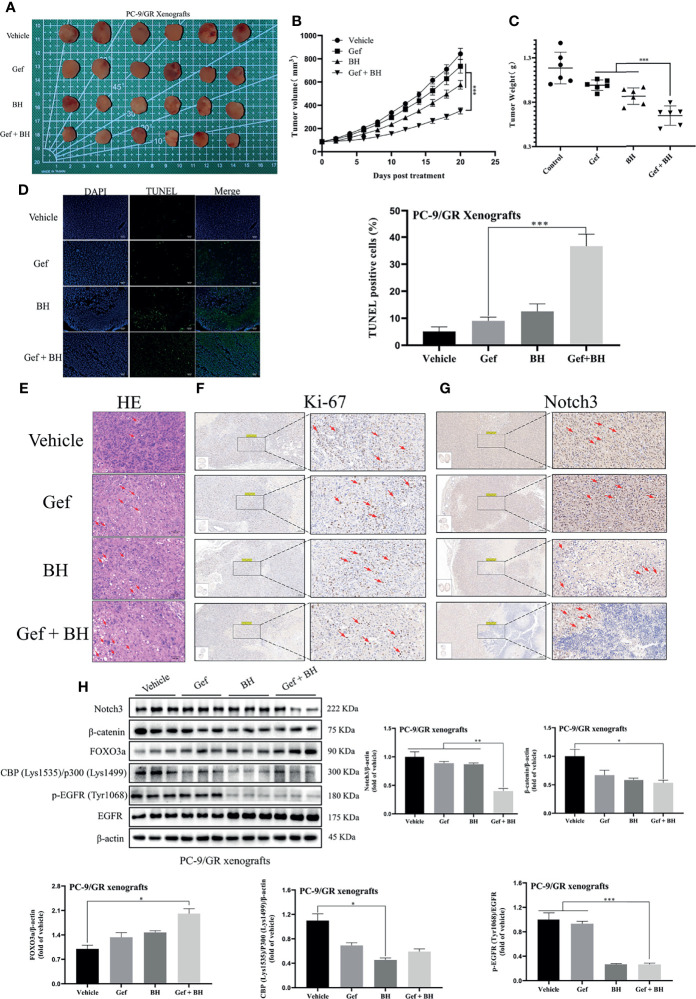
Combination of BH and gefitinib suppressed the growth of PC-9/GR xenografts tumor. **(A)** Representative pictures of tumors. *n* = 6 per group. **(B)** Tumor volume of PC-9/GR xenografts. **(C)** Tumor weights and size of PC-9/GR xenografts. **(D, E)** TUNEL assay and H&E staining were performed to examine histological morphology and apoptosis of tumor tissues, and the scale bar was 100 μm. **(F, G)** The protein levels of Ki-67 and Notch3 of PC-9/GR xenografts were detected by IHC, and the scale bar was 200 μm. **(H)** The expressions of Notch3, β-catenin, FOXO3a, p-EGFR (Tyr1068), EGFR, and BPC (Lys1535)/P300 (Lys1499) of PC-9/GR xenografts were measured by Western blot. “*” represents Gef +BH group, BH group, Gef group vs. the group (*p < 0.05, **p < 0.01, ***p< 0.001).

To further determine the therapeutic effect of BH combined with gefitinib, we established a nude mouse xenograft model of PC-9/GR cells. The results revealed that treatment with BH at 2.5 mg/kg alone or a low dose of gefitinib (25 mg/kg) alone showed a slight inhibitory effect on tumor growth of PC-9/GR by intragastric administration, respectively (**Figure 8A**), while co-administration of BH (2.5 mg/kg) and gefitinib (25 mg/kg) significantly suppressed xenograft tumor growth (**Figures 8B, C** **)**. There were no significant changes in body weights and HE staining of the heart, kidney, liver, lung, and spleen tissues (**Figures S4A, B**). In addition, BH and gefitinib combination treatment caused significant cancer cell apoptosis compared with a single treatment, increased significantly the number of TUNEL-positive cells in cancer tissues, and reduced the number of Ki-67-positive cells in cancer tissues (**Figures 8D–F** **)**. Also, IHC staining demonstrated that the level of Notch3 was inhibited by BH and gefitinib combination (**Figure 8G**). Western blotting further proved that the expression of Notch3, as well as β-catenin downstream genes CBP (Lys1535)/p300 (Lys1499) and p-EGFR (Tyr1068) in the tumor tissues, was reduced (**Figure 8H**). Taken together, combining a Notch3 inhibitor with gefitinib inhibits the growth of TKI-resistant lung cancer xenografts more effectively than either treatment separately.

To prove that BH had no significant toxicity *in vivo*, the organ (**Table S2**), blood routine (**Table S3**), and biochemical indexes (**Table S4**) of nude mice were determined. Compared with the model group or positive drug group, we did observe no significant changes in the coefficients of lung and spleen and in the levels of white blood cells, lymphocytes, and neutrophils in the whole blood and in the levels of total cholesterol, triglycerides, low-density lipoprotein, and high-density lipoprotein in the serum. As a result, we examined the preliminary pharmacokinetic parameters (**Table 1** **and** **Tables S5–S7** in the **Supplementary Material**) of BH after intravenous and oral administration in SD rats. The preliminary pharmacokinetic parameters for a single dose of the compound BH after intravenous (2 mg/kg) and oral administration (10 mg/kg) were as follows: the half-life (*T*_1/2_) was 9.694 and 3.353 h, maximum concentration (*C*_max_) was 477.4 and 146.6 ng/ml, and the area under the plasma concentration–time curve (AUC_0–last_) was 943.5 and 961.2 ng h/ml, respectively. The results showed that oral bioavailability (*F*) was 20.38%.

**Table 1:** Preliminary pharmacokinetic parameters for BH^a^.

Parameter	2 mg/kg iv	10 mg/kg po
Clearance (CL, L h^−1^ kg^−1^)	1.911	
Volume of distribution (*V*_ss_, L/kg)	26.52	
Half-life (*T*_1/2_, h)	9.694	3.353
Time of maximum concentration (*T*_max_, h)		1
Maximum concentration (*C*_max_, ng/ml)	477.4	146.6
AUC_0–last_ (ng h/ml)	943.5	961.2
AUC_0–inf_ (ng h/ml)	1,051.6	976.2
Oral bioavailability (*F*, %)		20.38

a Values are the average of three runs.

## Discussion

So far, lung cancer patients, with EGFR-TKI resistance, are difficult to be treated by targeted therapy (35), and the combination regimen will also be affected by adverse reactions and the patients’ physical conditions (36). Therefore, it is urgent to seek new targeted therapies to abate drug resistance in patients with NSCLC. **Figure 8** shows that we and others have reported that inhibiting EGFR potently induces β-catenin through a non-canonical Notch3-dependent mechanism (20), which induces the combination of β-catenin and FOXO3a gene, thus promoting the expression of alternative target genes (37). For example, FOXO3a acts as a transcriptional coactivator of β-catenin and enhances the expression of EGFR target genes (38). FOXO3a, a transcription factor of the forkhead family, is a key regulator of crucial proteins associated with cell cycle progression, apoptosis, proliferation, metabolism, and tumorigenesis (39, 40). Many studies showed that FOXO3a expression is identified as a potential biomarker for the diagnosis, prognosis, and treatment of multiple malignant tumors (41, 42). Interestingly, an integrated genomic approach revealed that low expression of FOXO3a is associated with drug resistance in lung cancer cells (43). These findings indicate that FOXO3a could be a potential target of chemotherapeutic drugs, and its activity may increase the chemosensitivity of cancer cells to agents such as gefitinib.

To this end, we screened a series of small molecules with antitumor activity from *B. javanica* and docked them with Notch3 and EGFR proteins. BH and BD were predicted to bind with the same sites on Notch3 HD domain and showed similar affinity in molecular docking analysis, while they also demonstrated similar antitumor effects in previous cell model studies. BH and BD strongly inhibit the survival, migration, and invasion of NSCLC cell lines and induce cell apoptosis. However, the sensitivity of BD to 16HBE and LO2 cell lines is significantly higher than that of BH. In this study, the hub gene of EGFR-TKI drug persistence in EGFR mutants was screened on the basis of differentially expressed genes and WGCNA, in order to explore potential drug resistance targets. Through the comprehensive analysis of co-expression network construction, functional enrichment, and hub gene screening, six EGFR and Notch inhibition-related key genes related to EGFR-TKI drug persistence were screened. We further demonstrated that BH inhibited the proliferation of NSCLC subcutaneous mouse xenografts, and verified the expression of four proteins (NOTCH3, CTNNBIP1, EGFR, and FOXO3a) from the hub targets of the WGCNA and GEO co-expression network. In Western blotting and IHC analysis, BH significantly induced the expression of FOXO3a in tumor tissue, whereas BH downregulated the expression of FOXO3a upstream regulators (NOTCH3, β-catenin) and Bcl-2 downstream genes in tumor tissue. These results are consistent with the expression patterns analyzed in GEO and TCGA databases, showing that patients with high levels of FOXO3a are better able to overcome EGFR-TKI resistance and survive longer. Therefore, we further investigated the effect of brucine H on EGFR-TKI drug persistence of NSCLC through the Notch3/β-catenin signal pathway. We used the gefitinib-resistant NSCLC cell line PC-9/GR obtained from continuous exposure to gefitinib to examine the role of FOXO3a and the effect of BH. The protein level of β-catenin in PC-9/GR was higher than that in PC-9, but the reverse was found in FOXO3a. Furthermore, BH and gefitinib synergistically induced cell apoptosis in PC-9/GR and inhibited the protein levels of β-catenin, thus activating FOXO3a, but gefitinib alone could not. It suggested that BH may enhance the sensitivity of resistant cells to gefitinib through inhibition of β-catenin and FOXO3a activation. We then explored the therapeutic benefit of BH–gefitinib combination using a xenograft model of PC-9/GR cells. Gefitinib showed a slight inhibition of tumor growth, but BH–gefitinib together showed an important reduction in xenograft tumor growth. Further immunohistochemical staining and Western blotting showed that the combined therapy could increase the expression of FOXO3a and inhibit the expression of Notch3, β-catenin, and pY1068-EGFR. We speculated that inhibition of EGFR and activation of FOXO3a potently induce β-catenin *via* a Notch3-dependent mechanism, which promotes the sustained development of drugs in animal models and the early development of clinical drug resistance. Dual targeting of EGFR and Notch3 may have a greater impact on inhibiting TKI-resistant lung cancer cell xenograft growth than focusing on either pathway alone, and the transcription factor p300/CBP contains an acetyltransferase domain that directly interacts with Notch3 *via* modulation of acetylation to promote degradation of Notch3 (44), so P300/CBP levels may be a useful mechanism-related marker of Notch3 activity in these patients.

**Figure 9:**
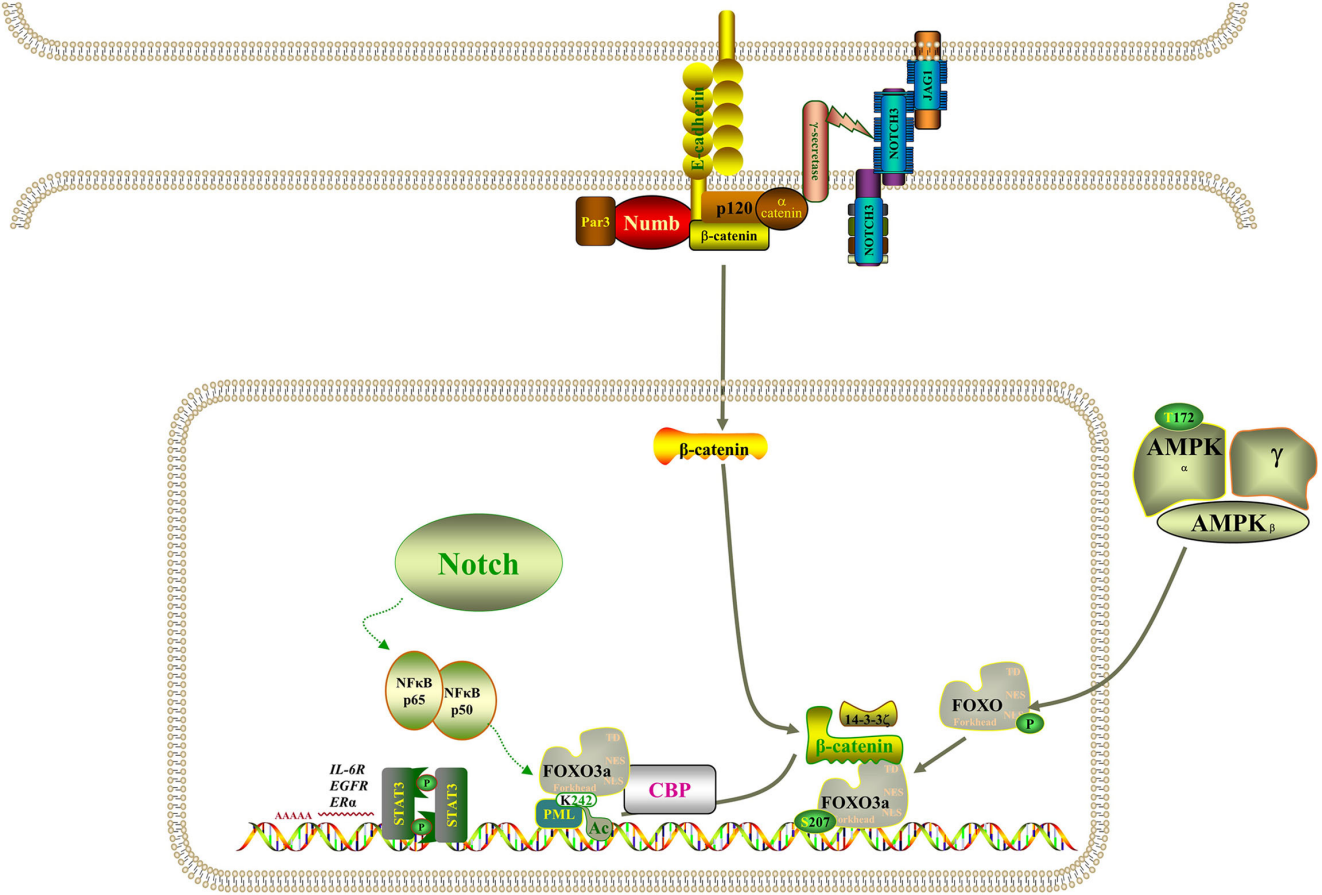
Model of the regulatory signaling networks of Notch3/β-catenin/FOXO3a in EGFR-TIK resistance and CSC properties of lung cancer.

However, the molecular mechanism of acquired drug resistance involves the combined action of multiple pathways and multiple targets, which is much more complicated than we expected. There is increasing evidence that Notch3 and other carcinogenic driving mechanisms will have synergistic effects, in which the irregular expression and/or mutation of Notch3, in combination with other mutations, likely results in a more serious phenotype and more resistance to treatment (45). It has been demonstrated that activation of β-catenin activity leads to tumor proliferation and reduced mouse survival in xenograft models, that EGFR tyrosine kinase inhibitor can activate β-catenin and Notch3 by inhibiting EGFR signal, and that knocking out Notch3 reduces β-catenin activity and resistance to targeted drugs (20, 46, 47). In addition, β-catenin correlates negatively with FOXO3a expression, and FOXO3a knockdown resulted in significantly higher expression levels of β-catenin (48). Based on evidence that the Wnt–β-catenin and Notch3 pathways play a part in tumorigenesis and that there is an interaction between FOXO3a and β-catenin, we speculate that Notch3 controls the expression of FOXO3a through non-classical dependence on β-catenin in lung cancer cell resistance, thereby regulating EGFR signal. The study also found that the activity of Notch3 is also necessary in the evolution of the refractory treatment of EGFR mutant NSCLC, which has been identified as the substrate of EGFR-mediated tyrosine phosphorylation, and inhibiting EGFR activity will reduce tyrosine phosphorylation, thus enhancing Notch3 activity (21). Remarkably, Notch3 overexpression was also observed in KRAS-mutated lung adenocarcinoma cells and correlated positively with aldehyde dehydrogenase (ALDH) expression, resulting in enhanced CSC phenotype (49, 50). Furthermore, activation of NF-κB through miR-155 has been reported to downregulate FOXO3a, which brought about the acquisition of gefitinib resistance in patients with NSCLC that carried EGFR mutations (43). On the other hand, when the signal dynamics model of leukemia T cells was set up, it was observed that Notch3 could activate NF-κB by associating with the pTα chain of the pre-T-cell receptor (51). As shown in **Figure 9**, Notch signaling may also act through nuclear NF-κB on FOXO3a. It is clear from all the evidence that not only Notch3 contributes to the occurrence and development of non-small cell lung cancer but also related proteins within its pathway are involved in drug resistance and recurrence.

## Conclusions

Based on our study results, we concluded that BH substantially suppressed Notch3 signaling in human lung cancer, resulting in powerful antitumor activity in human lung cancer both *in vitro* and *in vivo*, and that it can improve the sensitivity of resistant NSCLC to gefitinib. In the meantime, these data establish new insights into the functional interaction among EGFR, Notch3, β-catenin, and FOXO3a: Notch3 is activated when EGFR-TIK inhibits EGFR and then stimulates β-catenin in a non-classical way, and β-catenin acts on EGFR in combination with FOXO3a; therefore, inhibition of Notch3 provides a potential combined treatment strategy for EGFR-TIK-resistant lung cancer.

## Data Availability Statement

The original contributions presented in the study are included in the article/**Supplementary Material**. Further inquiries can be directed to the corresponding authors.

## Ethics Statement

The animal study was reviewed and approved by Jiangxi University of Chinese Medicine Animal Care Committee (SCXK 2016-0006).

## Author Contributions

All authors contributed to the concept and design of the study. All authors contributed to the article and approved the submitted version.

## Funding

This work was supported by the National Natural Science Foundation of China (81860684), Jiangxi Natural Science Foundation (GJJ190689, 20212ACB206007), National Key R&D Program of China (2019YFC1712302), and Construction of First-Class Discipline of TCM Pharmacy in Jiangxi Province (JXSYLXKZHYAO016).

## Conflict of Interest

The authors declare that the research was conducted in the absence of any commercial or financial relationships that could be construed as a potential conflict of interest.

## Publisher’s Note

All claims expressed in this article are solely those of the authors and do not necessarily represent those of their affiliated organizations, or those of the publisher, the editors and the reviewers. Any product that may be evaluated in this article, or claim that may be made by its manufacturer, is not guaranteed or endorsed by the publisher.
