# Corrigendum: Bruceine H mediates EGFR-TKI drug persistence in NSCLC by Notch3-dependent β-catenin activating FOXO3a signaling

**DOI:** 10.3389/fonc.2024.1515205

**Published:** 2024-12-03

**Authors:** Jiahui Wu, Xiao He, Ziwei Xiong, Lingyu Shi, Daofeng Chen, Yulin Feng, Quan Wen

**Affiliations:** ^1^ Pharmacy, Jiangxi University of Chinese Medicine, Jiangxi, China; ^2^ National Pharmaceutical Engineering Center for Solid Preparation in Chinese Herbal Medicine, Jiangxi, China; ^3^ Pharmacy, Fudan University of Pharmacy, Shanghai, China

**Keywords:** bruceine H, Notch3 inhibitor, EGFR-TKI, acquired resistance, non-small cell lung cancer

In the published article, there was an error in **Figure 2D** as published. During the image combination process, the images of BH Control 2 and 3 were not deleted but were erroneously labeled as BD Control and BH 4 µM, respectively, resulting in an overall incorrect layout. The corrected **Figure 2D** and its caption “Matrigel invasion assays of A549 cells were measured” appear below.

**Figure 2:**
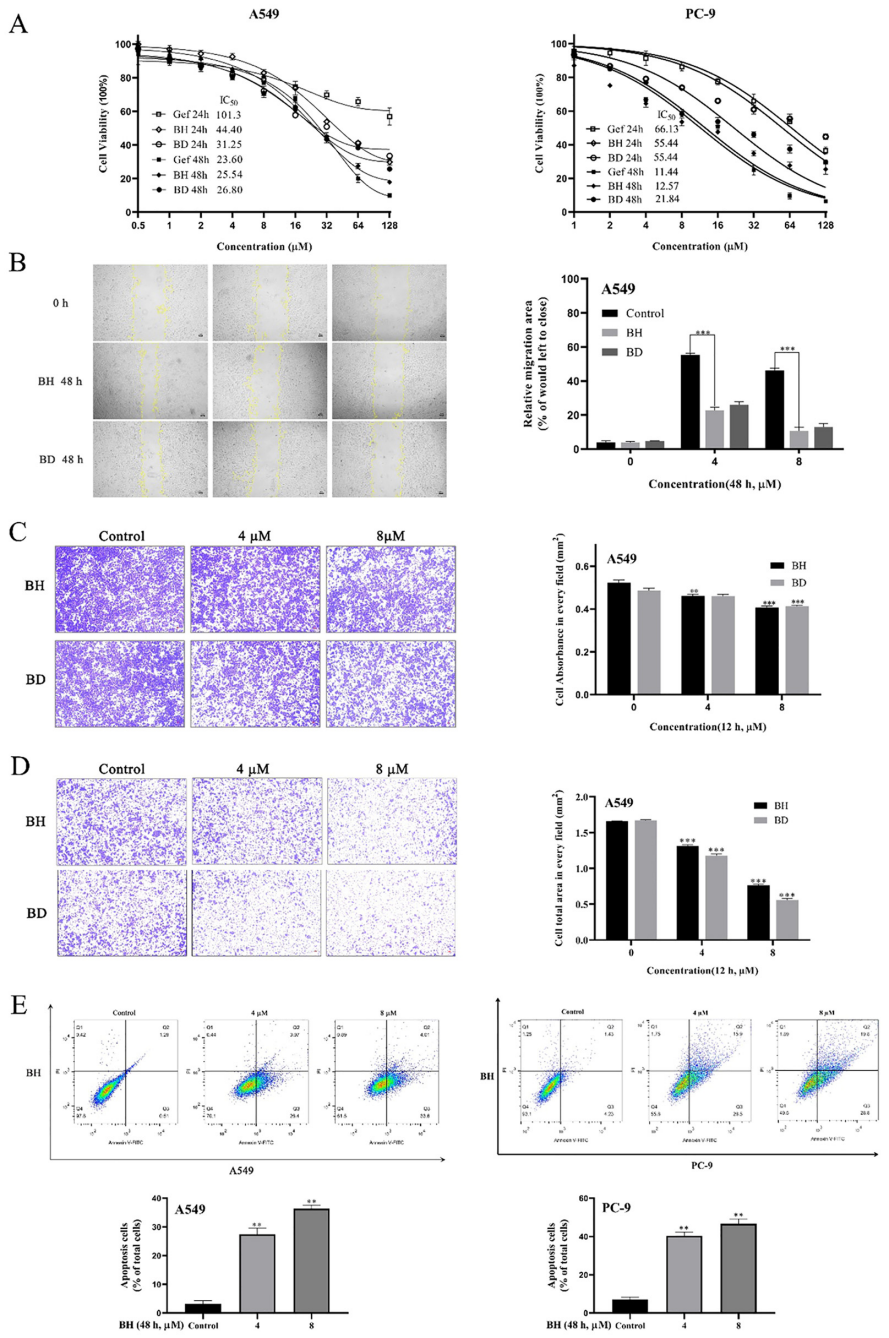
Antitumor effects of BH and BD against non-small cell lung cancer (NSCLC) cell lines *in vitro*. **(A)** The proliferation of cells as well as the IC50 was determined in A549 and PC-9 cells after 24 and 48 h. **(B)** Cell migration of A549 cells was detected by wound healing assay. **(C, D)** Transwell migration and Matrigel invasion assays of A549 cells were measured. **(E)** The apoptosis of A549 and PC-9 cells was measured using flow cytometry 48 h after treatment with BH. All experiments were independently conducted at least three times and showed representative data. “**” represents BH group vs. the Control group (**p < 0.01, ***p < 0.001).

The authors apologize for this error and state that this does not change the scientific conclusions of the article in any way. The original article has been updated.

