# Supplementary material for: Bruceine H Mediates EGFR-TKI Drug Persistence in NSCLC by Notch3-Dependent β-Catenin Activating FOXO3a Signaling
Source: Front Oncol. 2022 Apr 8;12:855603. doi: 10.3389/fonc.2022.855603 (PMC9024338; doi:10.3389/fonc.2022.855603)
Supplement: Supplementary file 1 [file DataSheet_1.docx]

Supplementary Material
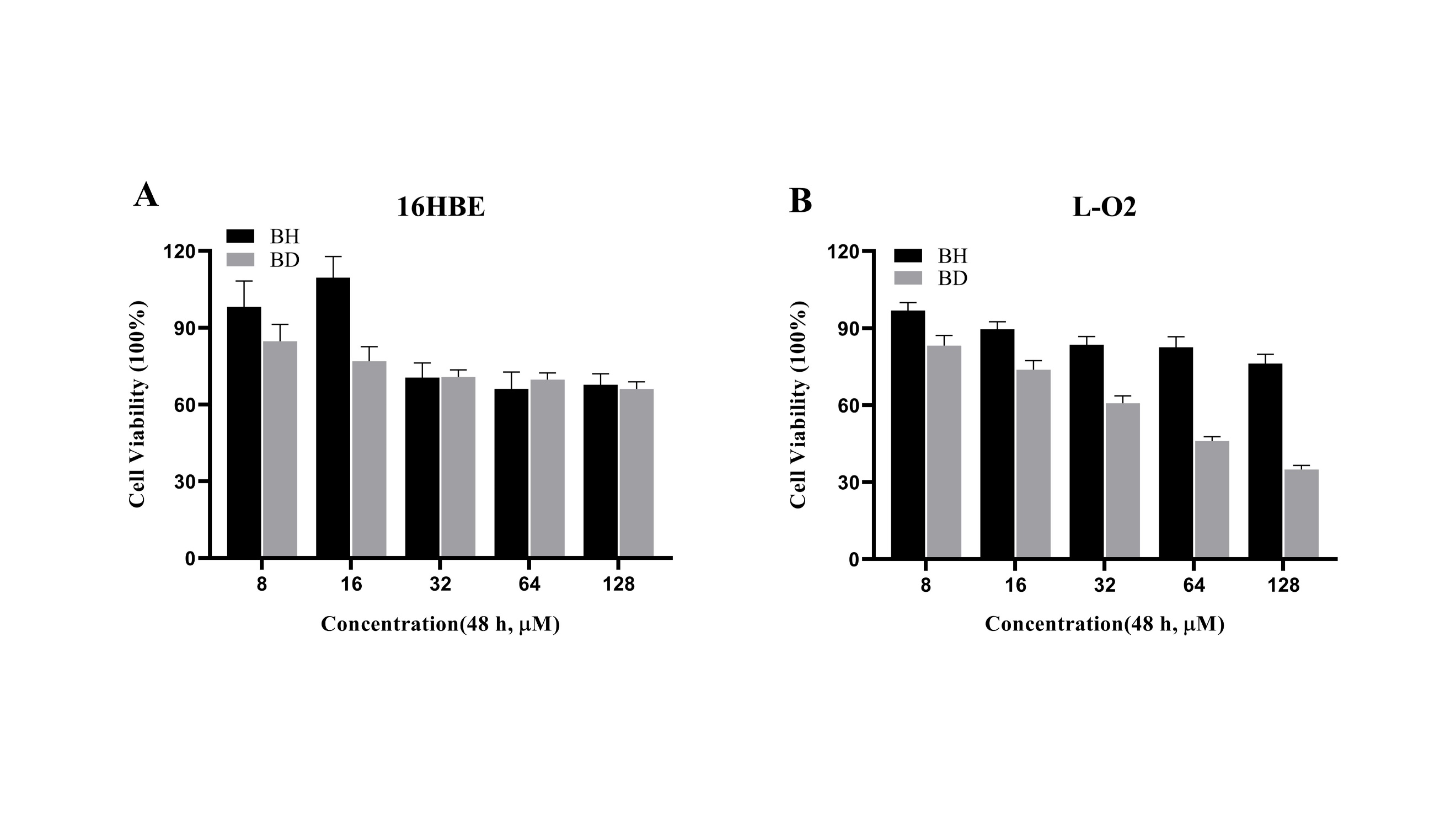


**Figure S1.** The toxicity of Bruceine H (BH) and Bruceine D (BD) to 16HBE **(A)** and LO2 **(B)** cells was evaluated.

**
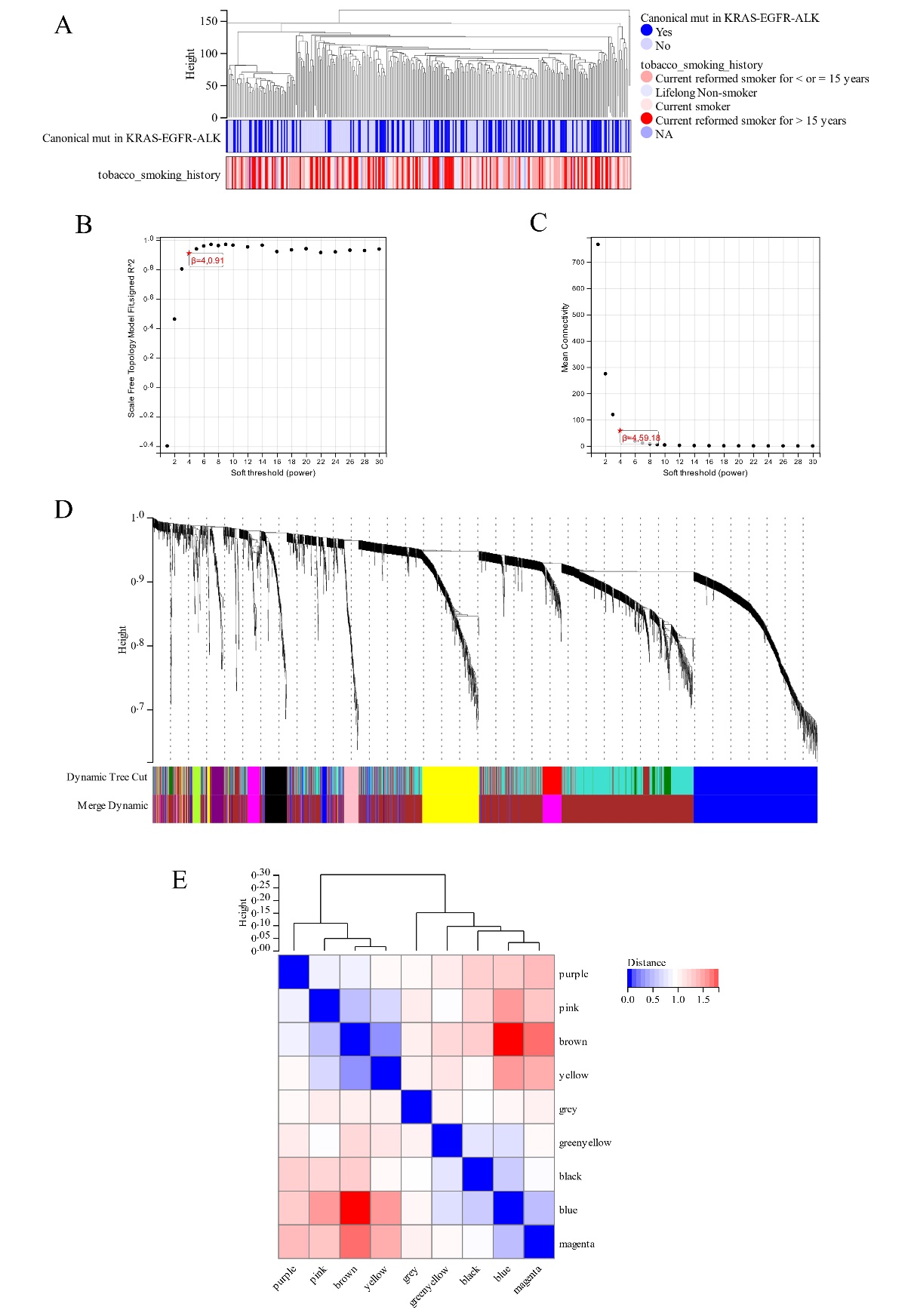
Figure S2.** Construction of gene co-expression modules. **(A)** Cluster analysis revealed the relationship between mutation and smoking history. **(B, C)** Analysis of network topology for various soft-thresholding powers. **(D, E)** Module eigengene dendrogram and eigengene adjacency heat map presented the relationship of the modules generated by the clustering analysis.

**
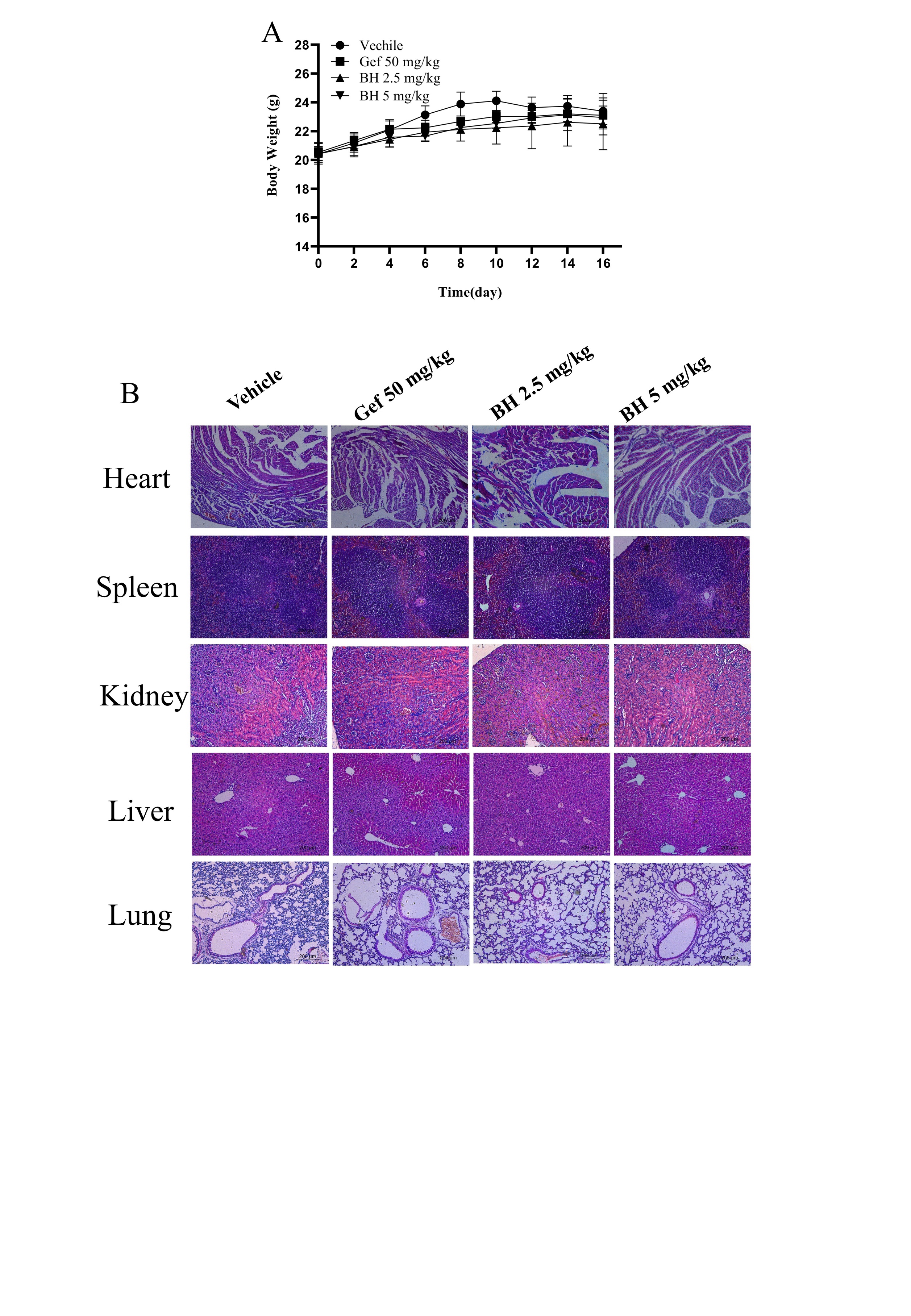
Figure S3.** Toxicity evaluation of BH in mice of A549 xenografts model. **(A)** Plots of body weight during days of treatment for mice; **(B)** HE staining of heart, spleen, kidney, liver and lung, of each group in A549 xenografts model.

**
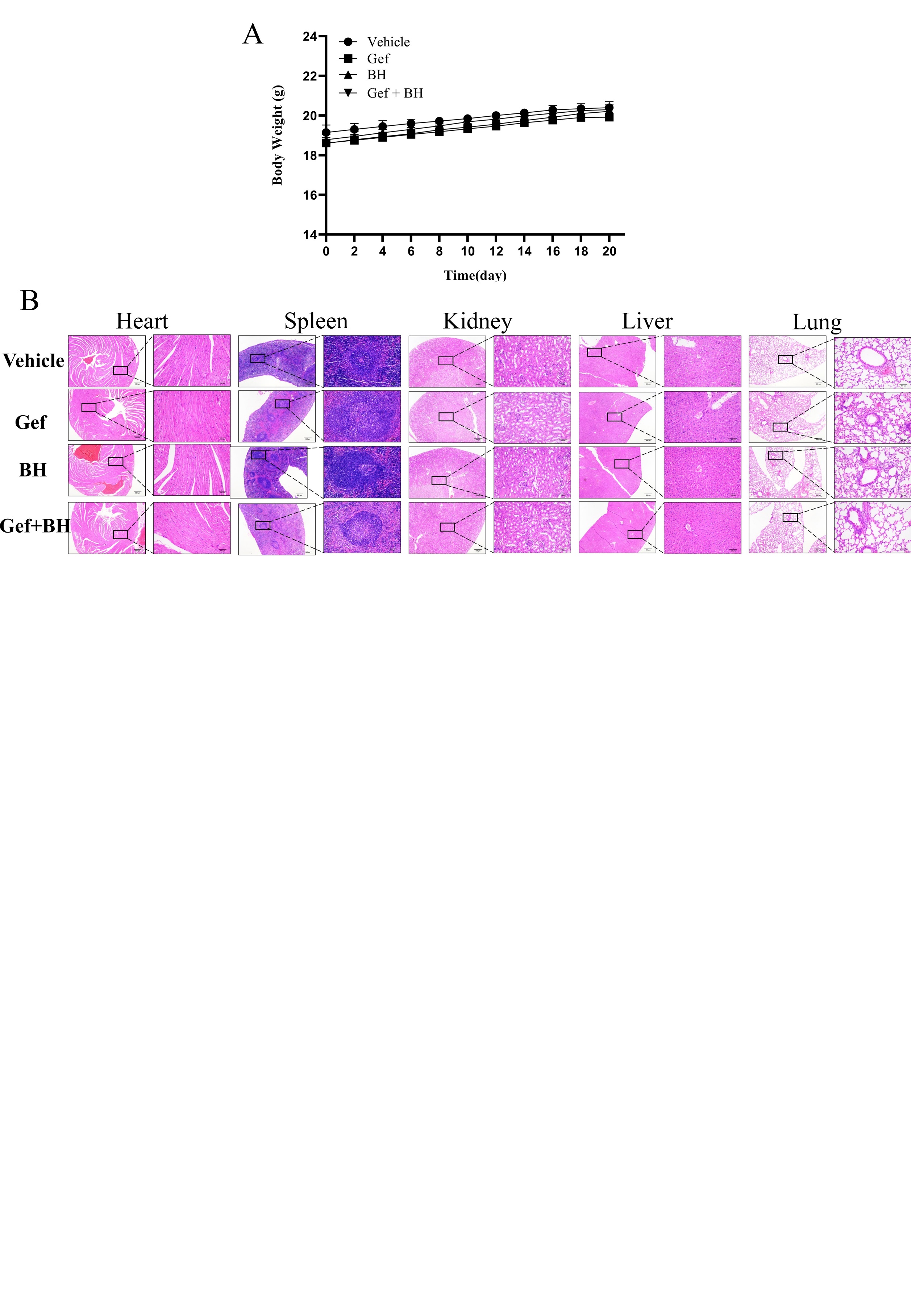
Figure S4.** Toxicity evaluation of combination of BH and gefitinib in PC-9/GR xenografts model. **(A)** Body weights of mice. **(B)** The size and appearance of heart, spleen, kidney, liver and lung of each group in PC-9/GR xenografts model.

| **Characteristics** | **Alive (N=618)** | **Dead (N=408)** | **Total (N=1026)** | **pvalue** |
| --- | --- | --- | --- | --- |
| **Cancer type** |  |  |  |  |
| lung | 618(60.23%) | 408(39.77%) | 1026(100.00%) |  |
| **Histological type** |  |  |  | 0.01 |
| lung adenocarcinoma | 334(32.55%) | 188(18.32%) | 522(50.88%) |  |
| lung squamous cell carcinoma | 284(27.68%) | 220(21.44%) | 504(49.12%) |  |
| **Canonical mut in KRAS** | **EGFR** | **ALK** |  | 0.14 |
| No | 83(32.05%) | 70(27.03%) | 153(59.07%) |  |
| Yes | 68(26.25%) | 38(14.67%) | 106(40.93%) |  |
| **Gender** |  |  |  | 0.08 |
| Female | 261(25.44%) | 149(14.52%) | 410(39.96%) |  |
| Male | 357(34.80%) | 259(25.24%) | 616(60.04%) |  |
| **Age, years** |  |  |  |  |
| Mean±SD | 65.68±9.19 | 67.21±9.59 | 66.29±9.38 |  |
| Median[min, max] | 67.00[33.00,87.00] | 68.00[40.00,90.00] | 67.00[33.00,90.00] |  |
| **Stage** |  |  |  | 1.10E-07 |
| Stage I | 355(35.01%) | 171(16.86%) | 526(51.87%) |  |
| Stage II | 166(16.37%) | 120(11.83%) | 286(28.21%) |  |
| Stage III | 77(7.59%) | 92(9.07%) | 169(16.67%) |  |
| Stage IV | 12(1.18%) | 21(2.07%) | 33(3.25%) |  |
| **Tobacco smoking history** |  |  |  | 0.15 |
| Current reformed smoker for < or = 15 years | 252(25.43%) | 170(17.15%) | 422(42.58%) |  |
| Current reformed smoker for > 15 years | 143(14.43%) | 76(7.67%) | 219(22.10%) |  |
| Current smoker | 142(14.33%) | 114(11.50%) | 256(25.83%) |  |
| Lifelong Non smoker | 60(6.05%) | 34(3.43%) | 94(9.49%) |  |

**Table S1.** The clinical information and sample size for TCGA LUNG dataset

**Table S2.** Effects of BH on nude mice organ (‾X±S)

| Group | Dose  mg×kg^-1^×d^-1^ | Lung index  (g/kg) | Spleen index  (g/kg) |
| --- | --- | --- | --- |
| Vehicle | - | 32.551±0.704 | 7.193±2.128 |
| Gef | 25 | 33.777±1.255 | 6.833±1.255 |
| BH | 2.5 | 32.463±2.078 | 5.949±0.526 |
| Gef + BH | 25 + 2.5 | 32.137±2.507 | 5.669±1.637 |

| Characteristic | Groups | | | | P |
| --- | --- | --- | --- | --- | --- |
|  | Vechine | Gef | BH | Gef + BH |  |
| TG[*^b^*](https://onlinelibrary.wiley.com/doi/10.1111/andr.12776#andr12776-note-0009_46) (mmol/L) | 1.08 ± 0.23 | 0.74 ± 0.16 | 0.70 ± 0.16 | 0.59 ± 0.05 | 0.2979 |
| TC[*^a^*](https://onlinelibrary.wiley.com/doi/10.1111/andr.12776#andr12776-note-0008_45) (mmol/L) | 2.87 ± 0.19 | 3.49 ± 0.56 | 3.25 ± 0.21 | 3.46 ± 0.64 | 0.7848 |
| HDL-C[*^b^*](https://onlinelibrary.wiley.com/doi/10.1111/andr.12776#andr12776-note-0009_46) (mmol/L) | 1.31 ± 0.24 | 0.74 ± 0.28 | 1.14 ± 0.18 | 0.61 ± 0.03 | 0.3879 |
| LDL-C[*^b^*](https://onlinelibrary.wiley.com/doi/10.1111/andr.12776#andr12776-note-0009_46) (mmol/L) | 1.13 ± 0.06 | 0.89 ± 0.45 | 1.40 ± 0.08 | 0.69 ± 0.09 | 0.5263 |

| Items | Groups | | | |
| --- | --- | --- | --- | --- |
|  | Vehicle | Gef | BH | Gef + BH |
| WBC (×10^8^/L) | 10.043±1.87 | 6.483±1.439 | 6.633±2.371 | 9.480±3.453 |
| LY (×10^9^/L) | 1.593±0.527 | 1.017±0.533 | 0.920±0.401 | 0.995±0.912 |
| MO (×10^9^/L) | 0.295±0.134 | 0.213±0.274 | 0.242±0.045 | 0.220±0.113 |
| GR (×10^9^/L) | 6.23±1.835 | 4.593±2.519 | 3.36±2.394 | 7.98±3.8074 |
| LY (%) | 15.667±2.312 | 14.2±2.685 | 14.7±6.165 | 16.6±0.849 |
| MO (%) | 3.7±1.386 | 5.067±1.582 | 5.043±1.086 | 4.8±0.707 |
| GR (%) | 82±2.052 | 83.467±4.674 | 80.567±11.274 | 90.3±0.849 |
| RBC (×10^12^/L) | 11.047±5.213 | 9.687±1.162 | 13.193±1.792 | 10.82±0.433 |
| HGB (g/L) | 176±20.000 | 148.667±17.616 | 172.333±12.014 | 165.333±10.263 |
| HCT (%) | 56±6.647 | 50.933±6.123 | 62.867±5.658 | 55.467±1.914 |
| MCV (fL) | 54.033±0.945 | 52.567±0.551 | 52.567±1.823 | 51.3±2.946 |
| MCH (Pg) | 16.067±0.764 | 15.367±0.306 | 15.567±0.289 | 15.3±0.755 |
| MCHC (g/L) | 297.667±9.018 | 292±2.646 | 296.333±10.970 | 298.333±15.695 |
| PLT (×10^9^/L) | 1094±163.887 | 934.333±117.1 | 1030±241.156 | 965±70.873 |
| MPV (fL) | 7.333±0.702 | 6.933±0.351 | 7.267±0.208 | 7.067±0.153 |
| PCT (mL/L) | 0.76±0.226 | 0.653±0.110 | 0.753±0.195 | 0.697±0.040 |

**Table S3.** Distribution of routine blood indicator in four groups of nude mice

**Table S4.** Lipid parameters among the four groups

Note

Data are means ± standard deviation. P values less than 0.05 were shown in bold.

Abbreviations: TG, triglycerides; TC, total cholesterol; LDL-C, low-density lipoprotein cholesterol; HDL-C, high-density lipoprotein cholesterol; non-HDL, TC-HDL-C; ANOVA, analysis of variance.

• a Value: pertain to statistically significant difference by one-way ANOVA.

• b Value: pertain to statistically significant difference by Kruskal-Wallis H test.

**Figure S5** and **Table S5-7.** Raw data for Preliminary Pharmacokinetic studies

**Figure S5.** The standard curve of BH was linear over the range in 5~1280 ng/mL (r=0.9957).

**Table S5.** Concentrations (ng/mL) of BH in rat plasma after p.o. administration of BH 10 mg/kg

| Time (h) | No1 | No2 | No3 | Mean | SD | RSD/% |
| --- | --- | --- | --- | --- | --- | --- |
| 0.25 | 13.6 | 10.5 | 11.3 | 11.8 | 1.609 | 13.6 |
| 0.5 | 68.4 | 55.4 | 50.6 | 58.133 | 9.209 | 15.8 |
| 0.75 | 110.8 | 98.5 | 104.2 | 104.5 | 6.155 | 5.9 |
| 1 | 154.4 | 145.3 | 140.1 | 146.6 | 7.238 | 4.9 |
| 2 | 130 | 117.7 | 120.4 | 122.7 | 6.465 | 5.3 |
| 4 | 114.8 | 99.4 | 89.6 | 101.267 | 12.703 | 12.5 |
| 6 | 97.6 | 75.3 | 68.5 | 80.467 | 15.222 | 18.9 |
| 8 | 50.4 | 35.4 | 41.2 | 42.333 | 7.564 | 17.9 |
| 12 | 16.8 | 10.4 | 22.1 | 16.433 | 5.859 | 35.7 |
| 24 | 2.8 | 1.8 | 5.4 | 3.333 | 1.858 | 55.7 |

**Table S6.** Concentrations (ng/mL) of BH in rat plasma after i.v. administration of BH 2 mg/kg

| Time (h) | No1 | No2 | No3 | Mean | SD | RSD/% |
| --- | --- | --- | --- | --- | --- | --- |
| 0.083333333 | 478 | 484.6 | 469.6 | 477.4 | 7.518 | 1.6 |
| 0.166666667 | 420.3 | 455.4 | 450.6 | 442.1 | 19.031 | 4.3 |
| 0.25 | 370.8 | 398.5 | 384.2 | 384.5 | 13.852 | 3.6 |
| 0.5 | 228 | 245.3 | 234.1 | 235.8 | 8.774 | 3.7 |
| 0.75 | 172.4 | 177.7 | 180.4 | 176.833 | 4.07 | 2.3 |
| 1 | 124.8 | 135.4 | 119.6 | 126.6 | 8.052 | 6.4 |
| 2 | 103.6 | 95.3 | 98.5 | 99.133 | 4.186 | 4.2 |
| 4 | 58 | 55.4 | 51.2 | 54.867 | 3.431 | 6.3 |
| 6 | 37.6 | 40.4 | 42.1 | 40.033 | 2.272 | 5.7 |
| 8 | 29.2 | 18.8 | 25.4 | 24.467 | 5.262 | 21.5 |
| 12 | 20 | 12.3 | 21.3 | 17.867 | 4.864 | 27.2 |
| 24 | 5.6 | 6.3 | 10.2 | 7.367 | 2.479 | 33.7 |

**Table S7.** Pharmacokinetics parameters of BH after p.o and i.v. administration in rat

| Parameters | Unit | i.v. | | | | | p.o. | | | | |
| --- | --- | --- | --- | --- | --- | --- | --- | --- | --- | --- | --- |
|  |  | No1 | No2 | No3 | Mean | SD | No1 | No2 | No3 | Mean | SD |
| AUC _(0-t)_ | ng/mL*h | 965.209 | 883.761 | 981.633 | 943.534 | 52.413 | 1066.9 | 858.063 | 958.688 | 961.217 | 104.441 |
| AUC _(0-∞)_ | ng/mL*h | 1018.163 | 977.839 | 1158.837 | 1051.613 | 95.022 | 1068.64 | 858.682 | 1001.201 | 976.174 | 107.193 |
| R_AUC _(t/∞)_ | % | 94.8 | 90.4 | 84.7 | 89.967 | 5.064 | 99.8 | 99.9 | 95.8 | 98.5 | 2.339 |
| AUMC _(0-t)_ | h*h*ng/mL | 4816.632 | 3928.001 | 5496.865 | 4747.166 | 786.735 | 5831.875 | 4275.963 | 5902.131 | 5336.656 | 919.259 |
| AUMC _(0-∞)_ | h*h*ng/mL | 6587.862 | 7623.703 | 12803.767 | 9005.111 | 3330.253 | 5879.636 | 4292.741 | 7263.126 | 5811.834 | 1486.353 |
| MRT _(0-t)_ | h | 4.99 | 4.445 | 5.6 | 5.012 | 0.578 | 5.466 | 4.983 | 6.156 | 5.535 | 0.59 |
| MRT _(0-∞)_ | h | 6.47 | 7.796 | 11.049 | 8.438 | 2.356 | 5.502 | 4.999 | 7.254 | 5.918 | 1.184 |
| VRT _(0-t)_ | h^2 | 30.325 | 31.091 | 40 | 33.805 | 5.378 | 17.271 | 15.275 | 25.371 | 19.306 | 5.347 |
| VRT _(0-∞)_ | h^2 | 73.322 | 156.114 | 243.787 | 157.741 | 85.244 | 18.047 | 15.623 | 54.204 | 29.291 | 21.609 |
| λ_z_ | 1/h | 0.106 | 0.065 | 0.058 | 0.076 | 0.026 | 0.291 | 0.327 | 0.125 | 0.248 | 0.108 |
| C__last_ | ng/mL | 5.604 | 6.155 | 10.282 | 7.347 | 2.557 | 0.506 | 0.202 | 5.305 | 2.004 | 2.862 |
| t_1/2z_ | h | 6.548 | 10.592 | 11.943 | 9.694 | 2.807 | 2.385 | 2.122 | 5.553 | 3.353 | 1.91 |
| T_max_ | h |  |  |  |  |  | 1 | 1 | 1 | 1 | 0 |
| V_z_ | mL/kg | 18560.708 | 31260.167 | 29744.277 | 26521.717 | 6935.974 | 32202.018 |  |  |  |  |
| CL_z_ | mL/h/kg | 1964.323 | 2045.327 | 1725.868 | 1911.839 | 166.071 | 9357.684 |  |  |  |  |
| C_max_ | ng/mL |  |  |  |  |  |  | 145.3 | 140.1 | 146.6 | 7.238 |
| C_0_ | ng/mL | 543.621 | 515.672 | 489.401 | 516.231 | 27.114 |  |  |  |  |  |

**Extraction and separation**

The dried and crushed seeds of *B. javanica* (20 kg) were soaked in petroleum ether (3 × 100 L, 24 h) to give defatted seeds, which were then extracted exhaustively with 95% ethanol (3 × 100 L, 2 h) under reflux for three times. After removal of the solvent under vacuum, the obtained viscous concentrate (2 kg) was suspended in water (4.0 L) and then partitioned with petroleum ether (4 ×4.0 L), CH_2_Cl_2_ (4 × 4.0 L), EtOAc (4 × 4.0 L), n-BuOH (4 × 4.0 L) consecutively. The extracts were evaporated under vacuum to afford petroleum ether-soluble (500 g), CH_2_Cl_2_-soluble (265 g), EtOAc-soluble (280 g), and n-BuOH-soluble (550 g) extracts. The EtOAc-soluble extract was loaded on a silica gel column (3 kg, 200–300 mesh) and eluted sequentially with CH_2_Cl_2_/MeOH (100:1 to 0:1, v/v) to yield eight main fractions E1-E8, and Bruceine D (2.6 g) was crystallized from fraction E4; Fraction was subjected to repeated silica gel CC eluted with CH_2_Cl_2_/MeOH (80:1 to 0:1, v/v) and repeated by preparative HPLC (MeCN-H_2_O, 28:72) to obtain Bruceine H (280 mg).


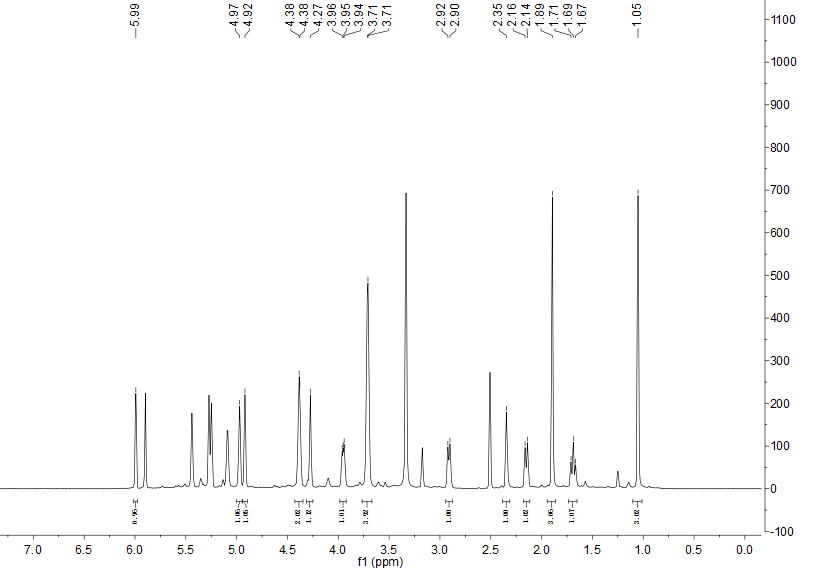


**Figure S6.** ^1^H NMR spectrum of BH

**Figure S7.** ^13^C NMR spectrum of
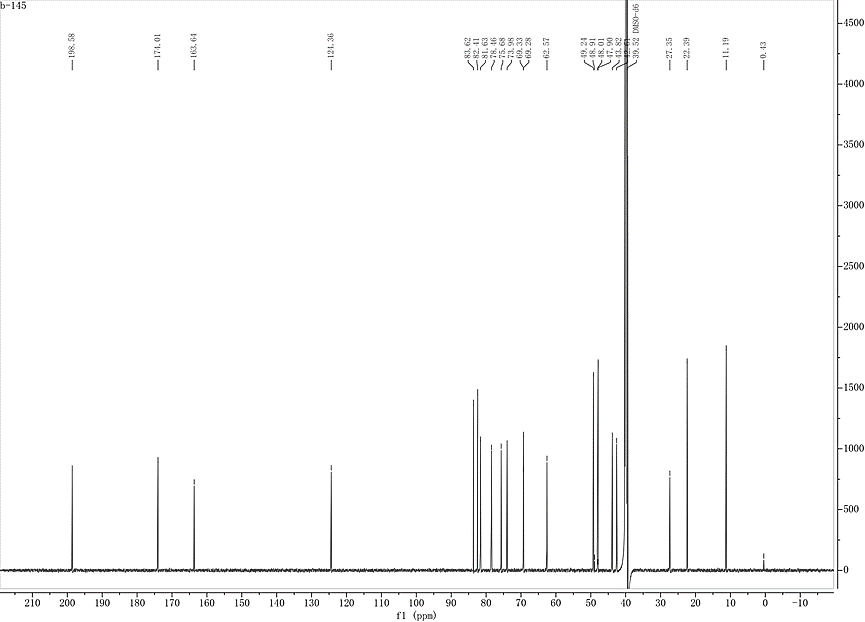
BH


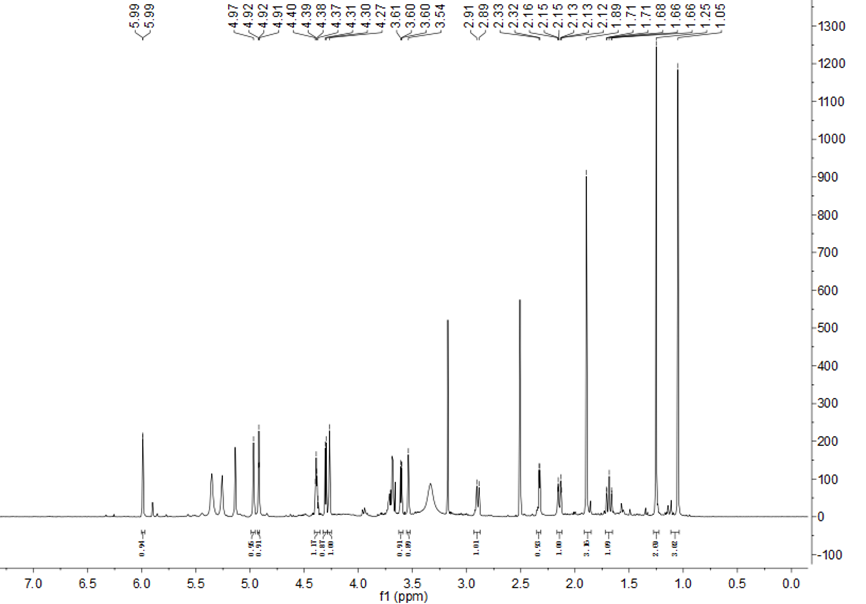


**Figure S8.** ^1^H NMR spectrum of BD

**
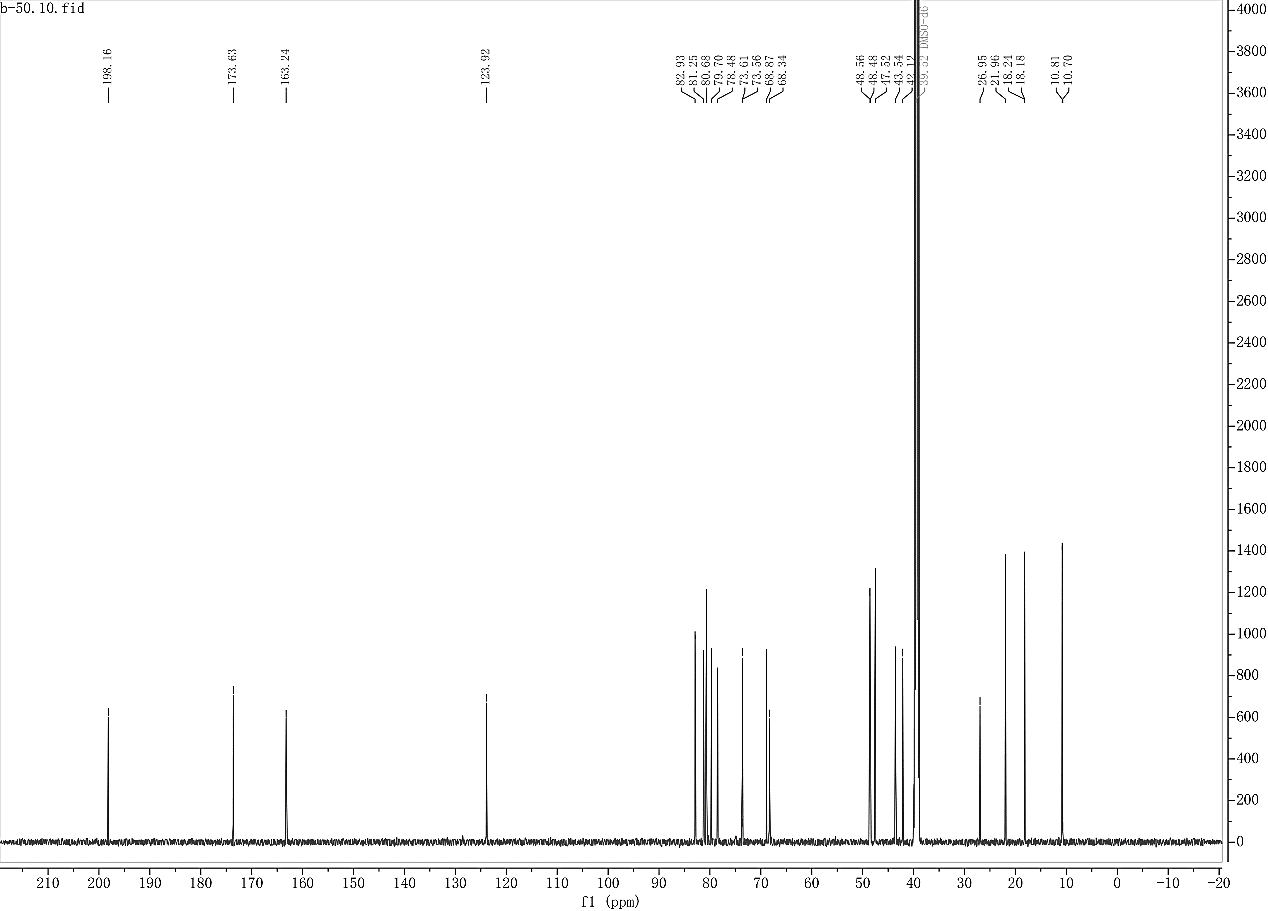
Figure S9.** ^13^C NMR spectrum of BD
