# Supplementary figures and images for: Bruceine H Mediates EGFR-TKI Drug Persistence in NSCLC by Notch3-Dependent β-Catenin Activating FOXO3a Signaling
Source: Front Oncol. 2022 Apr 8;12:855603. doi: 10.3389/fonc.2022.855603 (PMC9024338; doi:10.3389/fonc.2022.855603)

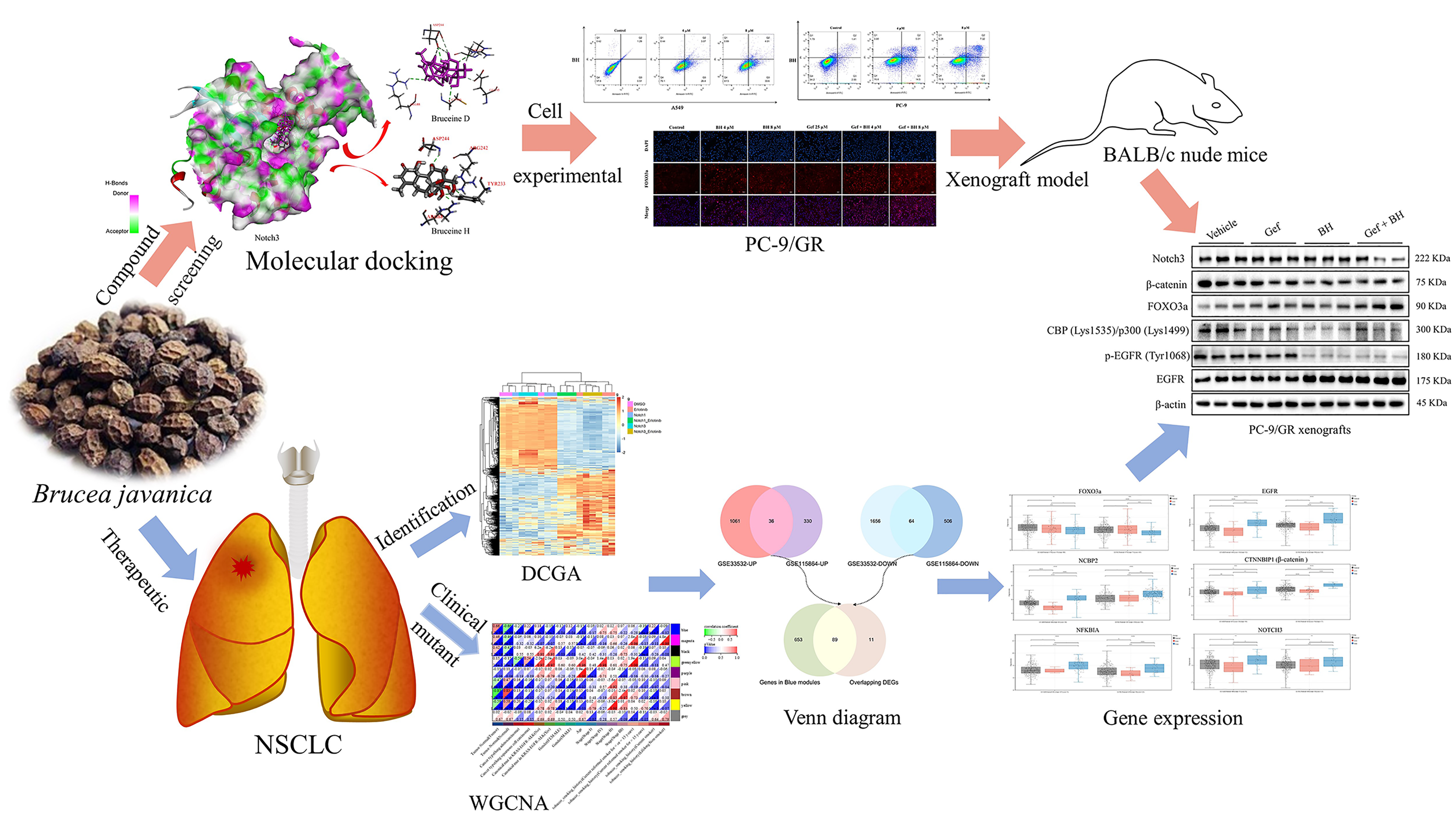

Supplement: Supplementary file 2 [file Image_1.tif]
